# The changing epidemiology of dengue in China, 1990-2014: a descriptive analysis of 25 years of nationwide surveillance data

**DOI:** 10.1186/s12916-015-0336-1

**Published:** 2015-04-28

**Authors:** Shengjie Lai, Zhuojie Huang, Hang Zhou, Katherine L Anders, T Alex Perkins, Wenwu Yin, Yu Li, Di Mu, Qiulan Chen, Zike Zhang, Yanzi Qiu, Liping Wang, Honglong Zhang, Linjia Zeng, Xiang Ren, Mengjie Geng, Zhongjie Li, Andrew J Tatem, Simon I Hay, Hongjie Yu

**Affiliations:** Division of Infectious Diseases, Key Laboratory of Surveillance and Early-warning on Infectious Disease, Chinese Center for Disease Control and Prevention, 155 Changbai Road, Changping District, Beijing, 102206 China; Department of Geography and Environment, University of Southampton, Southampton, SO17 1BJ UK; Oxford University Clinical Research Unit, Wellcome Trust Major Overseas Programme, 764 Vo Van Kiet, District 5, Ho Chi Minh City, Vietnam; Centre for Tropical Medicine, University of Oxford, Old Road Campus, Roosevelt Drive, Oxford, OX3 7FZ UK; Department of Epidemiology and Preventive Medicine, Monash University, Melbourne, Australia; Department of Biological Sciences and Eck Institute for Global Health, University of Notre Dame, Notre Dame, IN 46556 USA; Fogarty International Center, National Institutes of Health, Bethesda, MD 20892 USA; Flowminder Foundation, Roslagsgatan 17 SE-11355, Stockholm, Sweden; Spatial Ecology and Epidemiology Group, Tinbergen Building, Department of Zoology, University of Oxford, South Parks Road, Oxford, OX1 3PS UK

**Keywords:** Dengue, China, Import, Indigenous, Epidemiology, Outbreak

## Abstract

**Background:**

Dengue has been a notifiable disease in China since 1 September 1989. Cases have been reported each year during the past 25 years of dramatic socio-economic changes in China, and reached a historical high in 2014. This study describes the changing epidemiology of dengue in China during this period, to identify high-risk areas and seasons and to inform dengue prevention and control activities.

**Methods:**

We describe the incidence and distribution of dengue in mainland China using notifiable surveillance data from 1990-2014, which includes classification of imported and indigenous cases from 2005-2014.

**Results:**

From 1990-2014, 69,321 cases of dengue including 11 deaths were reported in mainland China, equating to 2.2 cases per one million residents. The highest number was recorded in 2014 (47,056 cases). The number of provinces affected has increased, from a median of three provinces per year (range: 1 to 5 provinces) during 1990-2000 to a median of 14.5 provinces per year (range: 5 to 26 provinces) during 2001-2014. During 2005-2014, imported cases were reported almost every month and 28 provinces (90.3%) were affected. However, 99.8% of indigenous cases occurred between July and November. The regions reporting indigenous cases have expanded from the coastal provinces of southern China and provinces adjacent to Southeast Asia to the central part of China. Dengue virus serotypes 1, 2, 3, and 4 were all detected from 2009-2014.

**Conclusions:**

In China, the area affected by dengue has expanded since 2000 and the incidence has increased steadily since 2012, for both imported and indigenous dengue. Surveillance and control strategies should be adjusted to account for these changes, and further research should explore the drivers of these trends.

Please see related article: http://dx.doi.org/10.1186/s12916-015-0345-0

**Electronic supplementary material:**

The online version of this article (doi:10.1186/s12916-015-0336-1) contains supplementary material, which is available to authorized users.

## Background

Dengue is an acute infectious disease caused by infection with any one of four serotypes of dengue virus (DENV 1-4), which are transmitted by *Aedes* mosquitoes [1]. There are an estimated 390 million dengue infections per year, of which 96 million manifest clinically (any level of disease severity) [2], among an estimated 2.5 to 4 billion people living in over 100 countries where DENV transmission occurs [1-4]. More than 70% of people at risk reside in the Asia Pacific region, making this region the global epicenter of dengue activity [2,5,6]. Susceptibility to dengue in humans is universal. Recovery from infection with one serotype confers lifelong homologous immunity, but only short-term protection against other serotypes, and sequential infections put people at greater risk for severe illness [7-9]. Because no effective vaccine for dengue is currently available, the effective protective measures are those that suppress vector populations and prevent exposure to *Aedes* mosquito biting [1,10-13].

In 1978 dengue fever reemerged in mainland China, in Foshan City of Guangdong province, after being absent for around 30 years [14]. Dengue became a notifiable disease on 1 September 1989 in China, partly in response to outbreaks of dengue fever, with cases of dengue hemorrhagic fever being reported sequentially in Hainan, Guangxi, Fujian, Zhejiang, and Yunnan provinces during the 1980s. All of these provinces are located in the southeast coastal regions or around the national border with Myanmar, Laos, and Vietnam in Southeast Asia [15-17]. Here we describe the magnitude and distribution of dengue in mainland China based on the notifiable reporting data, focusing on seasonal and geographical patterns from 1990 to 2014, and characteristics of imported and indigenous cases from 2005 to 2014, so as to identify high-risk areas and seasons and thereby help plan resource allocation for dengue prevention and control.

## Methods

### National dengue surveillance program

On 1 September 1989, dengue was made statutorily notifiable in China. Dengue cases are diagnosed according to the unified diagnosis criteria issued by the Chinese Ministry of Health, including clinically diagnosed and laboratory confirmed cases (see next section) [18-21]. All probable or laboratory confirmed cases are reported to the Chinese Center for Disease Control and Prevention (China CDC) in Beijing. Two datasets were used in this study. One includes dengue cases, aggregated by gender and 5-year age group, reported monthly between 1990 and 2004 by all provinces in mainland China, which includes 22 provinces, four municipalities, and five autonomous regions. The other consists of individual dengue cases reported by doctors within 24 hours of diagnosis to the online National Notifiable Infectious Disease Reporting Information System at the China CDC from 2005 to 2014. The individual data include gender, age, address, nationality, type of diagnosis, imported or indigenous case, serotype, hospitalization, date of illness onset, and various potential risk factors (see Additional file 1: Table S1). All the data used in this study were anonymized; the identity of any individual case cannot be uncovered.

### Case definition

Three editions of criteria/guidelines for dengue diagnosis issued by the Chinese Ministry of Health in 1988, 2001, and 2008 were successively used from 1990 to 2014 (see Additional file 2: Table S2) [19-21]. Dengue cases are classified as probable or confirmed based on whether they are clinically diagnosed or laboratory confirmed. Probable cases are those diagnosed by local experienced physicians according to cases’ epidemiologic exposure and clinical manifestations; confirmed cases are clinically diagnosed cases for which any of the following laboratory results are reported by the local public health institutes: fourfold or greater increase in DENV-specific IgG antibody titer between paired samples, or positive DENV polymerase chain reaction (PCR) test, or positive virus isolation and identification [19-21]. Before 1 September 2008, a DENV-IgM positive laboratory result was classified as a confirmed case, but since then has been classified as probable. In the notifiable disease database, dengue cases are not reported with information about their disease severity, and classification as either a probable or confirmed case was not recorded before 2005.

At the provincial level, an imported case of dengue is defined as a dengue case for which the patient had traveled to a dengue-affected foreign country or province of mainland China, and reported being bitten by mosquitoes within 15 days of the onset of illness [22,23]. In some cases, importation is defined based on laboratory results showing that the infecting dengue virus had a high sequence similarity in the preM/E region compared with viruses isolated from the putative source region where the patient had traveled [23]. Otherwise, a dengue case is considered to be an indigenous case. All imported cases in our datasets were classified as importations either from other countries or from other provinces. A determination about whether a case in the individual-level dataset from 2005 to 2014 was imported or indigenous was made by local public health institutes, following epidemiological investigations after a dengue case was diagnosed and reported by local physicians.

### Data analysis

We included all cases with illness onset from 1 January 1990, to 31 December 2014 in the analysis. The crude incidence rate was estimated as the number of probable and confirmed cases divided by the population at each year-end, which was extracted from the China population and employment statistics yearbook 2013 of the National Bureau of Statistics of China. The population data in 2014 were estimated from the population data and growth rates in 2013. The epidemiologic characteristics of imported and indigenous cases in China from 2005-2014 were also summarized. The Kruskal-Wallis test was used to examine whether the median age was significantly different between imported and indigenous cases, with a significance level of α = 0.05.

To analyze the time series of dengue cases, we created heat maps of the proportion of cases reported in each month from 1990 to 2014 by province, standardized by the total number of cases in each province over the 25-year period, and ordered by latitude of capital city of each province (see Additional file 3: Figure S1 and Additional file 4: Table S3). To compare seasonal patterns of dengue by imported and indigenous cases, we also created heat maps of the mean value of the proportion of cases in each week from 2005 to 2014. Version 3.0.1 of the *R* statistical software (R Foundation for Statistical Computing, Vienna, Austria) [24] was used to produce the graphs and heat maps and conduct statistical analyses, and *ArcGIS* 10.0 (ESRI, Redlands, CA, USA) was used to plot the geographical distribution of cases.

## Results

### Overall incidence

**Figure 1:**
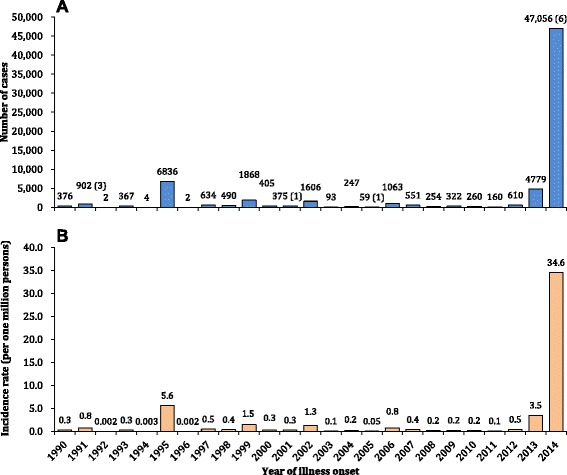
The incidence of dengue cases reported in mainland China, 1990-2014 (N = 69,321). Panel **A**: The aggregated number of cases by year with the numbers of deaths in parentheses. Panel **B**: The morbidity of dengue per one million residents of mainland China at the end of each year.

During the 25-year period from 1990 to 2014, 69,321 cases of dengue including 11 deaths were reported to the national dengue surveillance system in China, with an average of 2.2 cases per one million residents each year in mainland China. Annual case numbers displayed striking variations, with the highest recorded in 2014 (47,056 cases) and the lowest in 1992 and 1996 (only two cases) (Figure 1).

**Figure 2:**
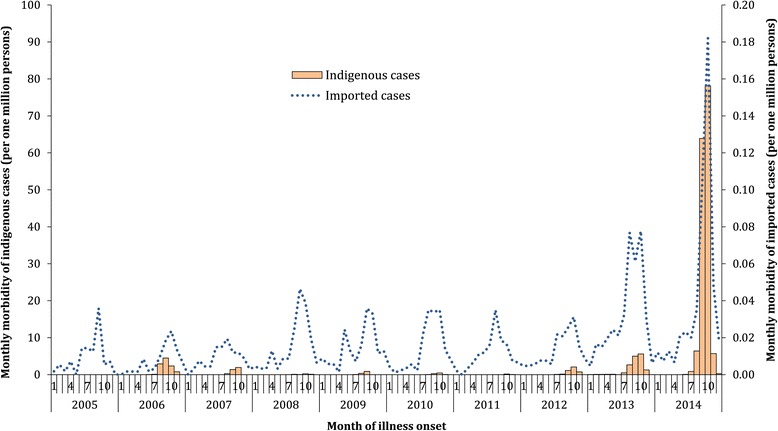
The morbidity of imported (N = 2,061) and indigenous (N = 53,053) dengue cases by month per one million residents of affected provinces at the end of each year in mainland China, 2005-2014.

During 2005-2014, 55,114 cases including 7 deaths were reported, of which 2,061 (3.7%) were imported and 53,053 (95.3%) were indigenous (see Additional files 5, 6, and 7: Tables S4-S6). The annual incidence rate of imported cases was relatively stable, with a median of 0.2 case per one million residents of affected provinces per year (IQR: 0.1-0.2 cases/1,000,000), except for a slight increase in 2013 (0.4 case/1,000,000) and 2014 (0.5 case/1,000,000) (Figure 2). Indigenous cases were reported each year from 2006 to 2014, with a median annual incidence of 2.5 cases per one million residents of affected provinces (IQR: 0.6-9.1 cases/1,000,000), decreasing from 2006 to 2011, and increasing from 2012 to 2014 with a peak of 155.3 cases/1,000,000 and 6 deaths in 2014 (Figure 2 and see Additional file 8: Figure S2).

### Demographic and virologic features

**Figure 3:**
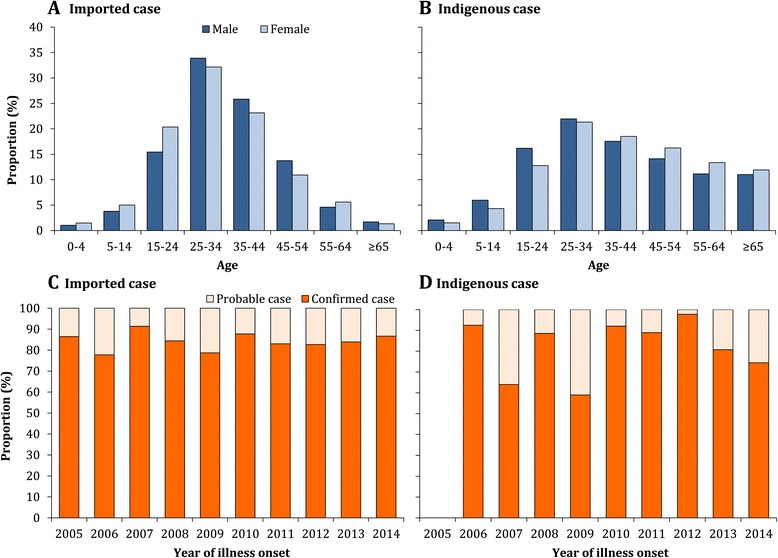
The age and gender distribution and proportion of imported (N = 2,061) and indigenous (N = 53,053) dengue cases that were laboratory confirmed by year, 2005-2014. Panel **A**: The age distribution of male and female imported cases. Panel **B**: The age distribution of male and female indigenous cases. Panel **C**: The proportion of imported cases that were laboratory confirmed each year. Panel **D**: The proportion of indigenous cases that were laboratory confirmed each year.

The overall male-to-female ratio was even from 1990 to 2014. However, there was a strong male predominance (2:1) among imported cases during 2005-2014 and an almost equal gender distribution for indigenous cases. The age distribution differed significantly between imported and indigenous cases during 2005-2014 (Kruskal-Wallis statistic = 228.3, *df* = 1, *P* < 0.001), with a younger median age of 32.5 years (IQR: 25.6-42.0) for imported cases and an older median age of 39.0 years (IQR: 26.3-53.7) for indigenous cases (Figure 3A and B, and see Additional file 9: Figure S3).

During 2005-2014, 75.8% (41,783/55,114) of reported dengue cases were laboratory confirmed; 84.7% (1,746/2,061) of imported cases and 75.5% (40,037/53,053) of indigenous cases. Of cases without laboratory confirmation, 89.4% were reported in 2014 (Figure 3C and D). Data on serotypes were only available for 415 (0.8%) indigenous cases during 2005-2014: 362 (87.2%) cases with DENV-1 in Guangdong during 2011-2014, 40 (9.6%) DENV-2 in Guangdong during 2013-2014, and 13 (3.1%) DENV-3 in Zhejiang in 2009 and Guangdong during 2012-2013. Among 18 (0.9%) imported cases with serotype data, all four serotypes were reported: DENV-1 (11 cases), DENV-2 (2), DENV-3 (3), and DENV-4 (2) during 2009-2014 (see Additional file 10: Figure S4). More demographic and epidemiologic results are shown in the Additional files.

### Geographical distribution

**Figure 4:**
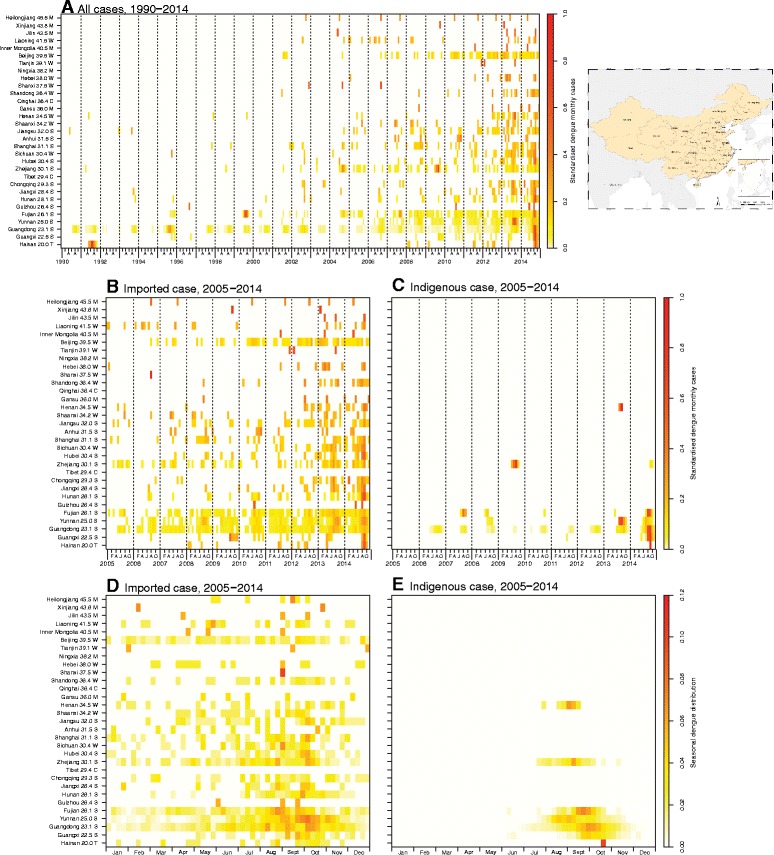
Heat map of dengue surveillance data by Chinese province, sorted by latitude of capital city, 1990-2014 (N = 69,321). On the Y-axis is listed the name of the province with the latitude of the capital city and a general classification of climate zone for each province. M: Mid-temperate; W: warm-temperate; C: cold; S: subtropical; T: tropical. A thumbnail map of all the provinces of China is provided at the end of the figure. Panel **A**: Time series of monthly dengue cases, 1990-2014, standardized by the number of total cases reported by each province. Panel **B**: Time series of monthly imported dengue cases, 2005-2014, standardized by the number of total cases reported by each province. Panel **C**: Time series of monthly indigenous dengue cases, 2005-2014, standardized by the number of total cases reported by each province. Panel **D**: Seasonal distribution of imported dengue cases, plotted as the mean value of the proportion of cases in each week of the year from 2005 to 2014. Panel **E**: Seasonal distribution of indigenous dengue cases, plotted as the mean value of the proportion of cases in each week of the year from 2005 to 2014.

**Figure 5:**
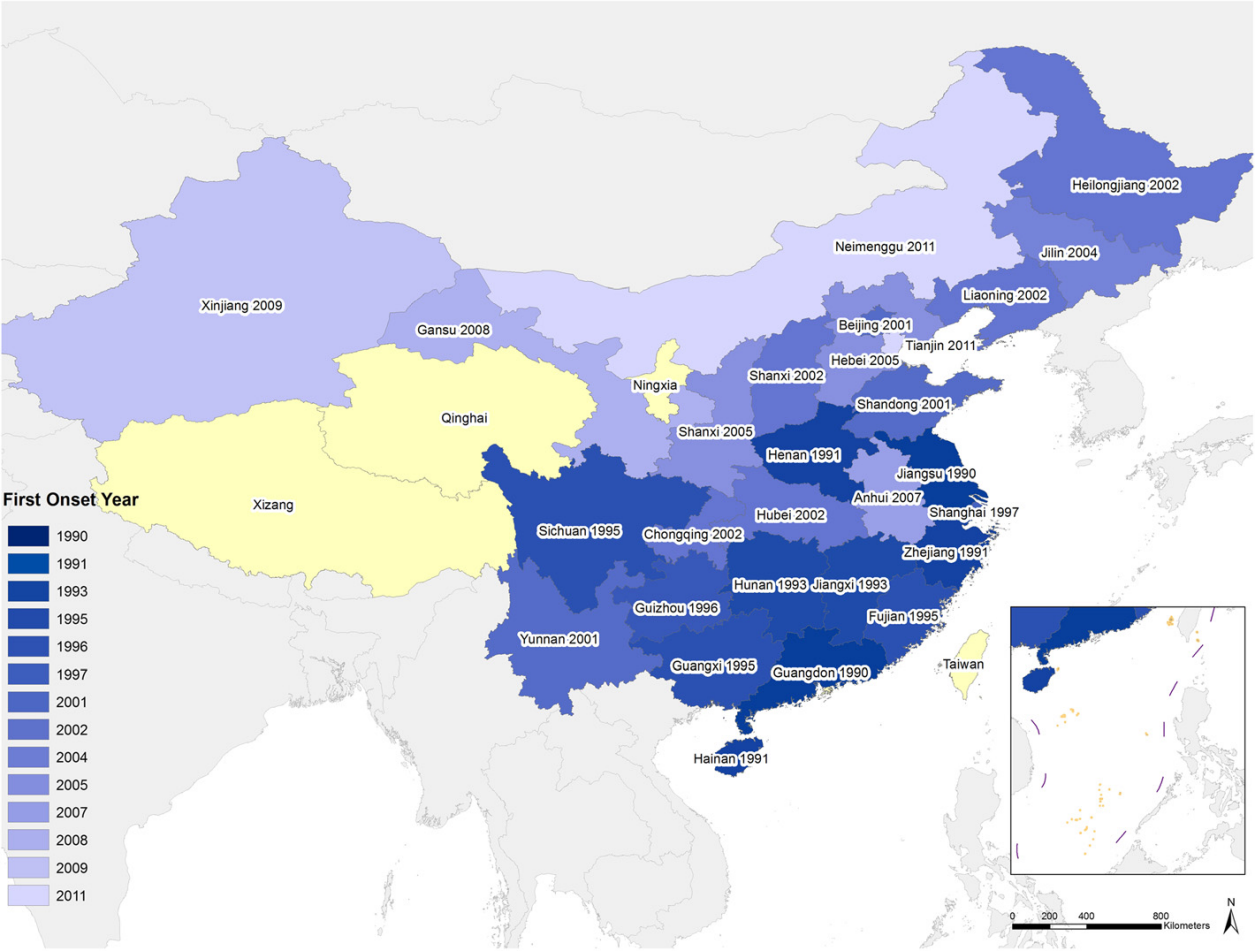
Years in which the first case of dengue was reported in each province in the time period of our dataset, 1990-2014.

The number of provinces reporting dengue cases has increased since 1990, from a median of 3 provinces per year (range: 1 to 5 provinces) during 1990-2000 to a median of 14.5 provinces per year (range: 5 to 26 provinces) during 2001-2014. The provinces affected have also expanded geographically from the southern to the northern parts of China (Figure 4A). During 2005-2014, except for Ningxia, Qinghai, and Tibet, all the other 28 provinces in mainland China had imported cases; the top provinces were Yunnan (28.8% of all imported cases), Guangdong (18.3%), Fujian (11.2%), Zhejiang (6.4%), and Hunan (5.4%) in southern China and the municipality of Beijing (4.4%) in northern China (Figure 4B and Figure 5). The suspected country of origin was recorded for 1,488 (81.5%) of all 1,826 dengue cases imported from other countries: 82.7% came from Southeast Asia, 8.3% from South Asia, and 5.6% from Africa. There were 235 cases exported from four domestic provinces of mainland China to other provinces: Guangdong (96.2%), Yunnan (2.1%), Guangxi (1.3%), and Hainan (0.4%). Of those interprovincial case movements, most (96.6%) occurred in 2014.

**Figure 6:**
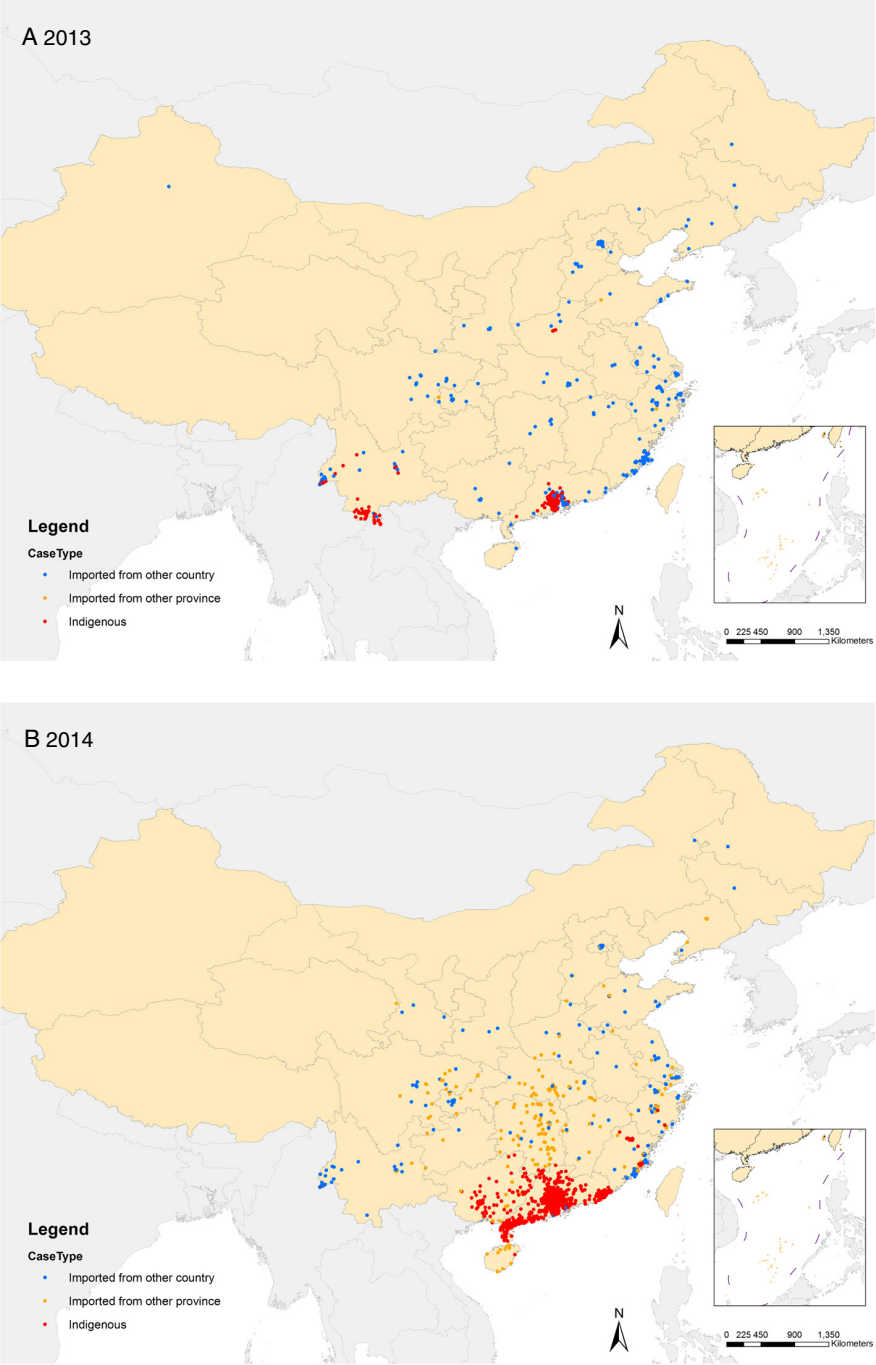
The geographic distribution of dengue cases in mainland China, 2013 and 2014. Panel **A**: The distribution of dengue cases in 2013 (N = 4,779). Panel **B**: The distribution of dengue cases in 2014 (N = 47,056).

During 2005-2014, all 53,053 indigenous cases were limited to just seven provinces: 94.3% were reported in Guangdong from 2006 to 2014, 3.0% in Yunnan (3 years), 1.6% in Guangxi (2 years), 0.7% in Fujian (5 years), 0.4% in Zhejiang (2 years), 0.05% in Henan in 2013, and 0.004% in Hainan in 2014. The affected regions expanded gradually over the 10-year period, from the coastal provinces (Hainan, Guangdong, Fujian, and Zhejiang) of southern China and provinces (Guangxi and Yunnan) adjacent to Southeast Asian countries to the central provinces of China (Henan) (Figures 4C, 5, 6 and see Additional file 11: Figure S5).

### Seasonality

During 2005-2014, 74.5% of imported cases were reported between July and November with a peak in October (24.6%) (Figures 2 and 4D). Generally, there was a subpeak of imported cases before the epidemic of indigenous cases each year, with a median lag of 2 months (IQR: 1-3 months) from the peak of imported cases to the month of the first indigenous case onset. Except for one indigenous case that occurred in April of 2010 in Guangdong, no indigenous cases were reported from January to May during 2005-2014, and 99.8% of indigenous cases occurred in the July to November period, peaking in September (40.4%) and October (48.6%). However, indigenous cases in the provinces with higher latitudes (Henan, Zhejiang, and Fujian), which were limited in their warm season duration, showed earlier peaks and shorter epidemic periods than the provinces at lower latitudes, such as Guangdong and Yunnan (Figure 4E).

## Discussion

In this study, a longitudinal surveillance dataset spanning 25 years in China was used to investigate changes in the epidemiological characteristics of imported and indigenous dengue during the period of dramatic social-economic changes that has occurred in China over the last three decades. We found that the geographic distribution of provinces affected by imported and indigenous dengue has expanded, especially since 2000, and recently the incidence rate of indigenous dengue has increased dramatically with a peak in the most recent recorded year.

### Magnitude and geographic extent of indigenous dengue

The incidence of dengue in China during the period 1990-2014 was lower than the epidemics in 1980 and 1985-1986, which resulted in more than 600,000 cases with 475 deaths overall in Hainan [17,25]. However, since 1990, indigenous dengue has not been limited to Hainan and Guangdong provinces, but has spread gradually from southern coastal tropical or subtropical regions (Guangdong, Guangxi, Hainan) to the neighboring northern and western regions (Fujian, Zhejiang, and Yunnan), and even to the central part of China - Henan province with a generally warm climate (Figure 5) [15,16,26]. Compared to the major epidemic in the 1980s, Hainan showed a dramatically decreased incidence of dengue with a few indigenous cases reported only in 1991 and 2014. Guangdong had the highest incidence of indigenous dengue over the last 25 years, with cases reported each year since 1997. Dengue transmission has also become evident in some previously unaffected areas, such as Ningbo city in the north of Zhejiang in 2004, Yiwu city in the inland part of Zhejiang in 2009, the central region of Henan province in 2013, and Nanping city in the central region of Fujian in 2014 [27-29]. This highlights the fact that the geographic range of dengue has apparently expanded in China, which is valuable information for consideration in national planning on dengue prevention and outbreak response. If dengue does continue to expand in China, this will need to be acknowledged in control planning, which currently focuses on Guangdong, Hainan, and Yunnan provinces in south China.

However, the number of reported dengue cases might be influenced by the change of diagnosis criteria and case definitions, especially through the introduction of more sensitive and rapid laboratory tests between 1990 and 2014, which could result in an increased number of reported cases without increased transmission. Compared to the 1988 criteria, the 2001 edition introduced the enzyme-linked immunosorbent assay (ELISA), immunofluorescence method, and dengue blot for serologic testing, RT-PCR for nucleic acid detection, and monoclonal antibody immunofluorescence for antigen detection. Then, the 2008 edition included MAC-ELISA for serologic testing and real-time fluorescence quantitative PCR for detecting nucleic acids, and classified a positive DENV-IgM result from a confirmed to a probable dengue case (see Additional file 2: Table S2) [19-21].

### Demographic characteristics of imported and indigenous cases

The age and gender distributions of imported and indigenous cases in China differ in a number of ways. Imported cases were younger than indigenous cases, and were more likely to be male. This may reflect a population of younger working male adults who tend to travel more domestically and regionally and thereby have more exposure risk to dengue. In addition, the indigenous cases occurred across all age groups, including the elderly, which is different from other countries in Southeast Asia where dengue is endemic and where most dengue cases occur in children or younger adults [30]. This pattern most likely is due to the fact that the population in China has very low seroprevalence of dengue antibodies, and is therefore broadly susceptible to dengue infection, whereas the population in dengue endemic countries has higher rates of immunity, especially in adults and the elderly [27,29,31]. However, the history of “mosquito bites” as part of the definition of imported case was impractical and likely introduced recall bias, which therefore probably underestimated the numbers and proportions of imported cases. A new guideline was issued in October 2014, which excludes “mosquitoes bite” in the definition of an imported case [23,32].

### Dengue and *Aedes* mosquitoes

*Aedes albopictus* has been found in nearly one third of China and is the most predominant species in south China except in Hainan province, which has both types of *Aedes* mosquitoes [16]. *Aedes aegypti* was implicated in outbreaks in Hainan in 1980 and 1985-1986 [17]. However, *Ae. albopictus* was the only vector species present in the outbreaks reported in Guangdong, Fujiang, and Zhejiang from 2004 to 2010 [27,33]. The importance of *Ae. albopictus* in dengue outbreaks appears to be increasing in China, which is worrisome because *Ae. albopictus* seems to adapt easily to new environments, even in a temperate climate, and to be associated with the huge population migration and urbanization in China and climatic change [16,34]. However, under a national sentinel vector surveillance project for dengue, only 16 counties out of 483 counties in the five provinces in south China conducted *Aedes* mosquito surveillance between June and October since 2005, and China did not have a national vector control program for dengue [19]. Therefore, it may be prudent for China to put more effort into mosquito surveillance and control for *Ae. albopictus.*

### Dengue virus serotypes

In this study, we found all four serotypes of dengue virus in dengue patients in China, all of which are capable of causing dengue of any clinical severity [16,30]. DENV-3 was the first serotype documented in Guangdong in 1978 [14] and in Hainan in 1980 [25]. Then, in 2009 and 2010, DENV-3 was isolated again in Guangdong from imported cases, but the 2010 outbreak was not a reemergence of the 2009 strain [35]. DENV-3 was also isolated during the outbreak in Zhejiang in 2009, in Yunnan in 2013, including from severe cases, and in the first outbreak in central China in 2013 [28,29,36]. DENV-1 has become the predominant serotype since the 1990s [27,37]. During 2005-2011, DENV-1 was the predominant serotype in circulation in Guangdong, while all four serotypes have been identified in indigenous patients from different outbreak localities since 2009 [31,38]. In addition, after an absence of 20 years since the DENV-4 outbreak in 1990, DENV-4 was detected during the outbreak in Guangzhou in 2010, in a Guangzhou resident who traveled back from Thailand [39]. DENV-2 was confirmed in Hainan in 1985-1986 [17], and a few cases were reported in 2013 and 2014. The increasing diversity in DENV strains imported to China, especially in 2013 and 2014, might increase the risk of DENV outbreaks and their severity in the near future, as well as the difficulty of dengue control. Therefore, monitoring this viral diversity should be considered in the design of surveillance and control strategies for China.

### Is dengue an endemic disease in China? Seasonality and virus source

Because of the geographic and seasonal restriction of cases, dengue in mainland China is still characterized as an imported disease and is not recognized as endemic [40]. This characterization rests on the assumption that imported cases play a key role in initiating outbreaks in China [27,41]. From this study, we have shown that imported cases were reported in nearly every month during 2005 to 2014. However, indigenous cases were mainly reported from July to November, which indicates a strong seasonality to dengue transmission in China, with peak transmission occurring mostly in the hot and humid seasons. Two factors are likely to contribute to this pattern. Firstly, the large amount of rainfall from July to October increases the availability of breeding habitats of mosquitoes, thereby causing increases in mosquito population densities and the potential for dengue transmission [42]. Secondly, transmission intensity can also fluctuate with temperature due to concomitant fluctuations in the length of the incubation period in the mosquito or mosquito mortality or blood feeding rates [43-45].

The dengue case data presented here represent only the clinically apparent infections which presented to health care facilities. Previous studies have shown that a large and variable proportion of DENV infections are clinically inapparent or mildly symptomatic [46,47], though adults are more likely to experience symptomatic illness than children [48]. This suggests that there is likely a larger pool of DENV infections and cases in China than is represented in this dataset. However, the overall incidence likely remains low compared to that in neighboring endemic countries [49,50].

In addition, most of the first local dengue outbreaks in each city and year can be traced back to imported cases that sparked the outbreaks [27,33,39]. Although for some outbreaks initial imported cases cannot be identified [28,37], the molecular fingerprints of strains often suggest that the outbreak is likely due to viruses imported from other countries [37]. Molecular epidemiological analysis in the last three decades also did not identify any new variants of viruses that are unique to mainland China [16]. Although DENV-1 was predominant in most years in Guangzhou city during 2001-2010, the strains from each year belonged to different genotypes and none of them was found to be predominant, though Southeast Asian countries were generally found to be the most likely source [38]. This suggests that dengue in China is due to localized transmission sparked by regular virus importations from returned travelers or visitors, rather than endemic transmission. Therefore, more attention should be directed toward the early identification of imported cases from other countries, especially from Southeast Asia.

### Limitations

There are some limitations in this study. Firstly, the data used were collected from passive public health surveillance. The data quality may be influenced by the key steps in surveillance including changing case definitions, reporting methods, availability of health facilities and laboratory diagnostics, under reporting, and completeness and accuracy of data over the years. Secondly, the individual case data were not reported before 2005, so demographic characteristics, laboratory confirmation, and the distribution of indigenous versus imported cases could only be analyzed from 2005-2014, and cases were not reported by the classification of disease severity.

### Challenge of dengue control in mainland China

The expansion of global air travel and seaborne trade, and the huge population movements in China overcome geographic barriers for both disease vectors and pathogens, enabling them to move great distances in short periods of time [51-53]. With the rapid growth of the economy and urbanization in China, more and more people in China have moved away from their original residences, especially from central China to coastal provinces, and from poor rural areas to urban centers [54]. This migration changes epidemiological dynamics and environments and can promote the transmission of dengue virus, increasing the population at risk of infection, and creating major challenges for prevention and control. Further, the increasing labor movements in and out of China to dengue endemic countries all over the world are driving changes in imported dengue dynamics.

The exceptionally high number of dengue cases in 2014 - a historical record since dengue became a notifiable disease in China in 1989 - serves as a reminder that even if dengue is not yet endemic in China, the possibility exists that the receptivity and vulnerability of certain areas to outbreaks could be increasing. Exploring the role of putative drivers of this huge outbreak, modeling and mapping the risk of importation and local transmission, and extracting lessons about how it could have been averted should be pursued immediately so as to inform future outbreak prediction and mitigation.

## Conclusions

Based on notifiable surveillance data in mainland China from 1990-2014, the area affected by dengue has expanded since 2000 and the incidence has increased steadily since 2012, for both imported and indigenous dengue. Surveillance and control strategies should be adjusted to account for these changes, and further research should explore the drivers of these trends.

### Ethical approval

It was determined by the National Health and Family Planning Commission, China, that the collection of data from dengue cases was part of continuing public health surveillance of a notifiable infectious disease and was exempt from institutional review board assessment.

## Additional files

Additional file 1: Table S1. The list of variables in the individual dataset of dengue cases from 2005 to 2014. Additional file 2: Table S2. Summary of diagnosis criteria and classification for dengue. Additional file 3: Figure S1. The general climate of each province in mainland China. The data is from the China Meteorological Administration (http://www.cma.gov.cn/). Additional file 4: Table S3. Summary of the geography and climate of each province in mainland China. Additional file 5: Table S4. Demographic and epidemiologic characteristics of dengue cases from 2005 to 2014. Additional file 6: Table S5. Demographic and epidemiologic characteristics of imported dengue cases by year from 2005 to 2014. Additional file 7: Table S6. Demographic and epidemiologic characteristics of indigenous dengue cases by year from 2005 to 2014. Additional file 8: Figure S2. The morbidity of imported (N = 2,061) and indigenous (N = 53,053) dengue cases in mainland China, 2005-2014. Panel A: The morbidity of imported cases per one million persons of affected provinces at each year-end. Panel B: The morbidity of indigenous cases per one million persons of affected provinces at each year-end. Additional file 9: Figure S3. The age distribution of imported (N = 2,061) and indigenous (N = 53,053) dengue cases by year and province. Panel A: The proportion of imported cases by age and year. Panel B: The proportion of indigenous cases by age and year. Panel C: The proportion of imported cases by age and top four provinces reported cases. Panel D: The proportion of indigenous cases by age and top four provinces reported cases. The number of cases is shown in parentheses in the legend. Additional file 10: Figure S4. The serotype distribution of imported (N = 18) and indigenous (N = 415) dengue cases by province, 2009-2014. Panel A: Serotype distribution of imported cases. Among 18 imported cases with serotype data, all four serotypes were reported: DENV-1 (11 cases), DENV-2 (2), DENV-3 (3), and DENV-4 (2) during 2009-2014. Panel B: Serotype distribution of indigenous cases. Data on serotypes were only available for 415 indigenous cases during 2005-2014: 362 (87.2%) cases with DENV-1 in Guangdong during 2011-2014, 40 (9.6%) DENV-2 in Guangdong during 2013-2014, and 13 (3.1%) DENV-3 in Zhejiang in 2009 and Guangdong during 2012-2013. Additional file 11: Figure S5. The geographic distribution of dengue cases by year in mainland China, 2005-2012.

## Abbreviations

CDC: Center for Disease Control and Prevention
DENV: dengue virus
ELISA: enzyme-linked immunosorbent assay
IQR: interquartile range
PCR: polymerase chain reaction
WHO: World Health Organization

## References

[1] World Health Organization: Dengue: guidelines for diagnosis, treatment, prevention and control. http://www.who.int/csr/resources/publications/dengue_9789241547871/en (2009). Accessed 15 November 2014. 23762963

[2] Bhatt S, Gething PW, Brady OJ, Messina JP, Farlow AW, Moyes CL (2013). The global distribution and burden of dengue. Nature..

[3] World Health Organization: Impact of dengue. http://www.who.int/csr/disease/dengue/impact/en. Accessed 27 November 2014.

[4] Brady OJ, Gething PW, Bhatt S, Messina JP, Brownstein JS, Hoen AG (2012). Refining the global spatial limits of dengue virus transmission by evidence-based consensus. PLoS Negl Trop Dis..

[5] Guzman MG, Harris E (2015). Dengue. Lancet..

[6] World Health Organization: Global strategy for dengue prevention and control 2012-2020. http://apps.who.int/iris/bitstream/10665/75303/1/9789241504034_eng.pdf. Accessed 15 November 2014.

[7] Halstead SB, O’Rourke EJ (1977). Dengue viruses and mononuclear phagocytes. I. Infection enhancement by non-neutralizing antibody. J Exp Med.

[8] Vaughn DW, Green S, Kalayanarooj S, Innis BL, Nimmannitya S, Suntayakorn S (2000). Dengue viremia titer, antibody response pattern, and virus serotype correlate with disease severity. J Infect Dis..

[9] Dejnirattisai W, Jumnainsong A, Onsirisakul N, Fitton P, Vasanawathana S, Limpitikul W (2010). Cross-reacting antibodies enhance dengue virus infection in humans. Science..

[10] Capeding MR, Tran NH, Hadinegoro SR, Ismail HI, Chotpitayasunondh T, Chua MN (2014). Clinical efficacy and safety of a novel tetravalent dengue vaccine in healthy children in Asia: a phase 3, randomised, observer-masked, placebo-controlled trial. Lancet..

[11] Wilder-Smith A (2014). Dengue vaccines: dawning at last?. Lancet..

[12] Villar L, Dayan GH, Arredondo-Garcia JL, Rivera DM, Cunha R, Deseda C (2015). Efficacy of a tetravalent dengue vaccine in children in Latin America. N Engl J Med..

[13] Achee NL, Gould F, Perkins TA, Reiner RC, Gubler DJ, Scott TW. A critical assessment of vector control for dengue prevention. PLoS Negl Trop Dis. 2015. In press. 10.1371/journal.pntd.0003655 PMC4423954 25951103

[14] Zhao HL, Luo QH, Shen G (1981). Epidemiology of the dengue outbreak in Shiwanzhen, Nanhai County, Guangdong Province [in Chinese]. Chin Med J..

[15] Fan WF, Yu SR, Cosgriff TM (1989). The reemergence of dengue in China. Rev Infect Dis..

[16] Wu JY, Lun ZR, James AA, Chen XG (2010). Dengue fever in mainland China. Am J Trop Med Hyg..

[17] Qiu FX, Chen QQ, Ho QY, Chen WZ, Zhao ZG, Zhao BW (1991). The first epidemic of dengue hemorrhagic fever in the People’s Republic of China. Am J Trop Med Hyg..

[18] Wang L, Wang Y, Jin S, Wu Z, Chin DP, Koplan JP (2008). Emergence and control of infectious diseases in China. Lancet..

[19] Ministry of Health of the People’s Republic of China (2008). Diagnostic criteria for dengue fever (WS 216-2008) [in Chinese].

[20] Ministry of Health of the People’s Republic of China (2001). Diagnostic criteria and principle of management of dengue fever (WS 216-2001) [in Chinese].

[21] Ministry of Health of the People’s Republic of China. Guidelines for diagnosis, treatment, prevention and control for dengue fever [in Chinese]. http://www.zaozhuang.gov.cn/art/2011/1/28/art_2330_352679.html (1988). Accessed 11 March 2015.

[22] Li Z, Yin W, Clements A, Williams G, Lai S, Zhou H (2012). Spatiotemporal analysis of indigenous and imported dengue fever cases in Guangdong province. China. BMC Infect Dis..

[23] Ministry of Health of the People’s Republic of China: Guideline of national dengue surveillance in China [in Chinese]. http://www.moh.gov.cn/uploadfile/2005818141858129.doc (2005). Accessed 5 January 2015.

[24] R Development Core Team (2010). R: A language and environment for statistical computing.

[25] Li FS, Yang FR, Song JC, Gao H, Tang JQ, Zou CH (1986). Etiologic and serologic investigations of the 1980 epidemic of dengue fever on Hainan Island. China Am J Trop Med Hyg..

[26] Wang Q, Xu Z, Dou FM, Zhou H, Wang XF, Yin WW (2009). Current situation and surveillance on dengue fever in China, 2005–2007 [in Chinese]. Zhonghua Liu Xing Bing Xue Za Zhi..

[27] Xu G, Dong H, Shi N, Liu S, Zhou A, Cheng Z (2007). An outbreak of dengue virus serotype 1 infection in Cixi, Ningbo, People’s Republic of China, 2004, associated with a traveler from Thailand and high density of Aedes albopictus. Am J Trop Med Hyg..

[28] Sun J, Lin J, Yan J, Fan W, Lu L, Lv H (2011). Dengue virus serotype 3 subtype III, Zhejiang Province, China. Emerg Infect Dis..

[29] Huang XY, Ma HX, Wang HF, Du YH, Su J, Li XL (2014). Outbreak of dengue fever in central China, 2013. Biomed Environ Sci..

[30] World Health Organization: Dengue fever and dengue hemorrhagic fever. http://www.who.int/mediacentre/factsheets/fs117/en (2009). Accessed 27 July 2014.

[31] Guo RN, Lin JY, Li LH, Ke CW, He JF, Zhong HJ (2014). The prevalence and endemic nature of dengue infections in Guangdong, South China: an epidemiological, serological, and etiological study from 2005-2011. PLoS One..

[32] Chinese Centers for Disease Control and Prevention. Guideline for dengue case surveillance [in Chinese]. http://www.chinacdc.cn/jkzt/crb/dgr/jszl_2235/201409/t20140929_104958.htm (2014). Accessed 11 March 2015.

[33] Peng HJ, Lai HB, Zhang QL, Xu BY, Zhang H, Liu WH (2012). A local outbreak of dengue caused by an imported case in Dongguan China. BMC Public Health..

[34] Rezza G (2012). Aedes albopictus and the reemergence of Dengue. BMC Public Health..

[35] Liang H, Luo L, Yang Z, Di B, Bai Z, He P (2013). Re-emergence of dengue virus type 3 in Canton, China, 2009-2010, associated with multiple introductions through different geographical routes. PLoS One..

[36] Zhang FC, Zhao H, Li LH, Jiang T, Hong WX, Wang J (2014). Severe dengue outbreak in Yunnan, China, 2013. Int J Infect Dis..

[37] Yang F, Guo GZ, Chen JQ, Ma HW, Liu T, Huang DN (2014). Molecular identification of the first local dengue fever outbreak in Shenzhen city, China: a potential imported vertical transmission from Southeast Asia?. Epidemiol Infect..

[38] Jiang L, Wu X, Wu Y, Bai Z, Jing Q, Luo L (2013). Molecular epidemiological and virological study of dengue virus infections in Guangzhou, China, during 2001-2010. Virol J..

[39] Jing QL, Yang ZC, Luo L, Xiao XC, Di B, He P (2012). Emergence of dengue virus 4 genotype II in Guangzhou, China, 2010: survey and molecular epidemiology of one community outbreak. BMC Infect Dis..

[40] Luo HM (2007). A big challenge for prevention and control of dengue fever in China [in Chinese]. South China Journal of Preventive Medicine..

[41] Chen SP (2011). The origin of dengue viruses caused the DF outbreak in Guangdong province, China, in 2006. Genet Evol..

[42] Sang S, Yin W, Bi P, Zhang H, Wang C, Liu X (2014). Predicting local dengue transmission in Guangzhou, China, through the influence of imported cases, mosquito density and climate variability. PLoS One..

[43] Chan M, Johansson MA (2012). The incubation periods of Dengue viruses. PLoS One..

[44] Brady OJ, Johansson MA, Guerra CA, Bhatt S, Golding N, Pigott DM (2013). Modelling adult Aedes aegypti and Aedes albopictus survival at different temperatures in laboratory and field settings. Parasit Vectors..

[45] Brady OJ, Golding N, Pigott DM, Kraemer MU, Messina JP, Reiner RJ (2014). Global temperature constraints on Aedes aegypti and Ae. albopictus persistence and competence for dengue virus transmission. Parasit Vectors..

[46] Endy TP, Anderson KB, Nisalak A, Yoon IK, Green S, Rothman AL (2011). Determinants of inapparent and symptomatic dengue infection in a prospective study of primary school children in Kamphaeng Phet. Thailand. PLoS Negl Trop Dis..

[47] Yoon IK, Rothman AL, Tannitisupawong D, Srikiatkhachorn A, Jarman RG, Aldstadt J (2012). Underrecognized mildly symptomatic viremic dengue virus infections in rural Thai schools and villages. J Infect Dis..

[48] Egger JR, Coleman PG (2007). Age and clinical dengue illness. Emerg Infect Dis..

[49] Simmons CP, Farrar J (2009). Changing patterns of dengue epidemiology and implications for clinical management and vaccines. PLoS Med..

[50] Cuong HQ, Vu NT, Cazelles B, Boni MF, Thai KT, Rabaa MA (2013). Spatiotemporal dynamics of dengue epidemics, southern Vietnam. Emerg Infect Dis..

[51] Tatem AJ, Hay SI, Rogers DJ (2006). Global traffic and disease vector dispersal. Proc Natl Acad Sci U S A..

[52] Messina JP, Brady OJ, Scott TW, Zou C, Pigott DM, Duda KA (2014). Global spread of dengue virus types: mapping the 70 year history. Trends Microbiol..

[53] Cowling BJ, Yu H (2015). Ebola: worldwide dissemination risk and response priorities. Lancet..

[54] Wayne Ma: Map Visualizes Chinese New Year Migration. 2014. http://blogs.wsj.com/chinarealtime/2014/01/27/map-visualizes-chinese-new-year-migration. Accessed 11 December 2014.

