# Supplementary material for: The changing epidemiology of dengue in China, 1990-2014: a descriptive analysis of 25 years of nationwide surveillance data
Source: BMC Med. 2015 Apr 28;13:100. doi: 10.1186/s12916-015-0336-1 (PMC4431043; doi:10.1186/s12916-015-0336-1)
Supplement: Additional file 1: Table S1. — The list of variables in the individual dataset of dengue cases from 2005 to 2014. [file 12916_2015_336_MOESM1_ESM.pdf]

Table S1. The list of variables in the individual dataset of dengue cases from 2005 to 2014.

Variables	Definition/classification	Completeness
ID	A unique 8-digital number for each case.	100% reported
Gender	Male and Female	100% reported
Age	The interval time from the date of birth to the date of onset	100% reported
Address	The living address (township level) of case when the case was recorded.	100% reported
Address code	A unique 8-digital number for each town	100% reported
Type of diagnosis	Probable case (clinical diagnosed case) and confirmed case (laboratory confirmed case)	100% reported
Serotype	DENV-I, II, III and IV (if applicable)	0.8% reported
Hospitalization	Inpatient or outpatient (Non-mandatory report)	30.9% reported
Nationality	Chinese or foreigner	100% reported
Type of case	Indigenous case, or case imported from other country, or case imported from other province in China	100% reported
Origin Country	The country where the case infected with dengue virus or had an exposure history during incubation period.	99.4% reported
Origin province of China	For case imported from other province in China, the province where the case infected with dengue virus or had an exposure history during incubation period.	100% reported
Date of onset	The date of illness onset	100% reported
Date of diagnosis	The date of diagnosis as a probable or confirmed dengue case	100% reported
Date of report	The first date of reporting to dengue surveillance system	100% reported
Date of Death	The date of case death, if applicable.	100% reported
