# Supplementary material for: The changing epidemiology of dengue in China, 1990-2014: a descriptive analysis of 25 years of nationwide surveillance data
Source: BMC Med. 2015 Apr 28;13:100. doi: 10.1186/s12916-015-0336-1 (PMC4431043; doi:10.1186/s12916-015-0336-1)
Supplement: Additional file 2: Table S2. — Summary of diagnosis criteria and classification for dengue. [file 12916_2015_336_MOESM2_ESM.pdf]

Table S2. Summary of diagnosis criteria and classification for dengue.

Variable	Guidelines for Diagnosis, Treatment, Prevention and Control for Dengue Fever	Diagnostic Criteria and Principle of Management of Dengue Fever	Diagnostic Criteria for Dengue Fever
**Issued by**	Chinese Ministry of Health	Chinese Ministry of Health	Chinese Ministry of Health
**Date issued**	20 June 1988	23 November 2001	28 February 2008
**Date enforced**	20 June 1988	1 May 2002	1 September 2008
**Epidemiologic linkage**	**1.1** Living in or travel to a dengue endemic country/region or presence at location with ongoing outbreak within previous 15 days of dengue-like illness.	**1.1** Living in or travel to a dengue endemic country/region or presence at location with ongoing outbreak within previous 15 days of dengue-like illness, and reported being bitten by mosquito within 5-9 days of illness onset.	**1.1** Travel to a dengue endemic country/region within previous 14 days of dengue-like illness<br>**1.2** Around the place of residence or place of work (e.g. 100m radius), there have been dengue case(s) within one month.
**Clinical description**	**2.1 Dengue fever (DF)**: Sudden onset, chills and fever (39-40°C within 24-36h, a small number of patients showed a biphasic fever). Headache, retro-orbital pain, joint pain, myalgia, arthralgia and lumbago, and a few patients develop abdominal pain. Fatigue and loss of appetite. Flushed skin on face, neck and chest, and conjunctival congestion, superficial lymphadenopathy, and tourniquet test positive. A total white blood cell and platelets counts are normal or decrease. Diversity-rash in limbs and trunk, some patients with bleeding tendency.<br>**2.2 Dengue hemorrhagic fever (DHF)**: a	**2.1** Sudden onset, chills and fever (39-40°C within 24-36h, a small number of patients showed a biphasic fever), with symptoms such as fatigue, nausea and/or vomiting.<br>**2.2** Aches and pains (e.g., headache, retro-orbital pain, joint pain, myalgia, arthralgia).<br>**2.3** Flushed skin on face, neck and chest, and conjunctival congestion.<br>**2.4** Superficial lymphadenopathy.<br>**2.5** Measles-like rash, scarlatiniform rash, and/or petechiae in the limbs, trunk, head and face in the course of illness (days 5-7); itching; no scaling; continued 3-5d.<br>**2.6** Encephalitis, encephalopathy, or	**2.1** Sudden onset, fever (39-40°C within 24-36h, someone shows biphasic fever); severe headache, retro-orbital pain, myalgia, arthralgia and fatigue; flushed skin on face, neck and chest, and conjunctival congestion, etc.<br>**2.2** Rash: measles-like rash, scarlatiniform rash, and/or needle-like hemorrhagic rash in the limbs, trunk, head and face in the course of illness (days 5-7); itching; no scaling; continued 3-5d.<br>**2.3** Bleeding tendency (tourniquet test positive): petechia, ecchymoses, purpura and injection site bleeding, or bleeding from the mucous membranes of mouth and nose,

Variable	Guidelines for Diagnosis, Treatment, Prevention and Control for Dengue Fever	Diagnostic Criteria and Principle of Management of Dengue Fever	Diagnostic Criteria for Dengue Fever
	dengue fever case develops the following symptoms: sporadic bleeding points in the limbs, face, armpits, mucous membrane after 2-3 days of illness onset, then become ecchymosis; bleeding of nose and mouth, bleeding in more than one organ of gastrointestinal tract, uterine, vaginal and urinary tract. Liver enlargement. Hematocrit increased more than 20%; Low platelets (< 100,000 cells per mm^3).<br>**2.3 Dengue shock syndrome (DSS)**: a dengue hemorrhagic fever case develops worse condition: clammy skin, restlessness, cyanotic lip, rapid and weak pulse, narrow pulse pressure ≤20mm Hg (2.7kPa) and undetectable in blood pressure, etc.	meningitis-like neurological disorders.<br>**2.7** Bleeding tendency (tourniquet test positive): occurs in the course of illness (days 5-8) with gingival bleeding, nose bleeding, gastrointestinal bleeding, subcutaneous hemorrhage, hematuria, hemoptysis, and vaginal bleeding, and/or chest and abdominal cavity bleeding, etc.<br>**2.8** Multiple organ bleeding.<br>**2.9** Liver enlargement.<br>**2.10** Shock.	gastrointestinal bleeding, hemoptysis, hematuria and vaginal bleeding in the course of illness (days 5-8).<br>**2.4** Massive hemorrhage of gastrointestinal tract, or chest and abdominal cavity bleeding, or intracranial hemorrhage.<br>**2.5** Liver enlargement, pleural or pericardial effusion.<br>**2.6** Shock syndrome: clammy skin, restlessness, rapid and weak pulse and narrow pulse pressure < 20mm Hg (2.7kPa) and undetectable in blood pressure, oliguria etc.
**Laboratory tests**	**3.1** Clinical routine tests of complete blood count, platelet, bleeding and clotting time; for severe cases, test hematocrit, and do additional tests according to the conditions.<br>**3.2** Cell culture isolation of dengue virus (DENV) by *Aedes albopictus* C6/36 cell, or 1-3 day-old newborn mice, or the larvae of Toxorhynchites.	**3.1** Thrombocytopenia (< 100x10^9/L). White blood cell count decrease, lymphocytes and mononuclear cell count increase.<br>**3.2** Hematocrit increased more than 20%.<br>**3.3** IgG anti-DENV positive in a serum specimen.<br>**3.4** IgM anti-DENV positive in a serum specimen.<br>**3.5** IgG anti-DENV ≥4-fold rise in titer in paired acute and convalescent serum specimens.	**3.1** A total white blood cell count decrease.<br>**3.2** Thrombocytopenia (< 100x10^9/L).<br>**3.3** Hemoconcentration (an increase in hematocrit ≥20% above average for age or a decrease in hematocrit ≥20% of baseline following fluid replacement therapy); hypoproteinemia.<br>**3.4** IgG or IgM anti-DENV positive in a serum

Variable	Guidelines for Diagnosis, Treatment, Prevention and Control for Dengue Fever	Diagnostic Criteria and Principle of Management of Dengue Fever	Diagnostic Criteria for Dengue Fever
	**3.3** Serologic tests positive: for paired acute and convalescent serum specimens, ≥4-fold rise in titer by complement fixation (CF), hemagglutination inhibition (HI), or neutralization test (NT); for a single acute phase serum specimen, 1:32 or higher in titer by CF, or 1:1280 or higher in titer by HI, or index ≥50 by NT.	The serologic tests included enzyme-linked immunosorbent assay (ELISA), HI, CF, immunofluorescence method (FA/IFA), Dengue blot (DB), and NT.<br>**3.6** Cell culture isolation of DENV by *Aedes albopictus* C6/36 cell or 1-3 day-old newborn mice; or detection of DENV nucleic acid by RT-PCR; or detection of antigens by monoclonal antibodies immunofluorescence (mbAb-FIA) in serum, cerebrospinal fluid (within 5 days of illness course), other body fluid or tissue.	specimen.<br>**3.5** Cell culture isolation of DENV by *Aedes albopictus* C6/36 cell or 1-3 day-old newborn mice in acute serum, cerebrospinal fluid, blood, or other tissue specimens.<br>**3.6** IgG anti-DENV ≥4-fold rise in titer in paired acute and convalescent serum samples.<br>The serologic tests included ELISA, mac-ELISA, HI, FA/IFA, NT.<br>**3.7** Detection of DENV nucleic acid by RT-PCR or real-time fluorescence quantitative PCR.
**Diagnosis and classification**	**4.1 Probable case:** a clinically compatible case of DF, DHF, or DSS with an epidemiologic linkage, as defined above.<br>**4.2 Confirmed case:** a probable case with a positive result of dengue virus isolation or serologic tests. The index case(s) of an outbreak or a new affected area should be a confirmed case.	**4.1 Suspected case**: a patient with item 1.1, 2.1 and 2.2, and one of item 2.3 to 2.7, as defined above.<br>**4.2 Probable case**: a suspected case with item 3.1 in a confirmed outbreak, or a suspected case with item 3.1 and 3.3 in an unconfirmed outbreak or presented as a sporadic case.<br>**4.3 Confirmed case:**<br>**DF**: a probable case with one of item 3.4, 3.5 and 3.6.<br>**DHF**: a confirmed DF case with item 2.8, 2.9 and 3.2.<br>**DSS**: a confirmed DHF case with item 2.10.	**4.1 Suspected case:** a patient with item 1.1 and 2.1, or a patient with item 2.1, 3.1 and 3.2, as defined above.<br>**4.2 Probable case:**<br>**DF**: a suspected case with 1.2, 3.1 and 3.2; or a suspect case with item 2.1, 3.1, 3.2 and 3.4<br>**DHF**: a probable case of DF with item 3.2, 3.3 and one of item 2.3 to 2.5.<br>**DSS**: a probable case of DHF with item 2.6.<br>**4.3 Confirmed case**: a probable case with one of item 3.5 to 3.7.
