# Supplementary figures and images for: The changing epidemiology of dengue in China, 1990-2014: a descriptive analysis of 25 years of nationwide surveillance data
Source: BMC Med. 2015 Apr 28;13:100. doi: 10.1186/s12916-015-0336-1 (PMC4431043; doi:10.1186/s12916-015-0336-1)

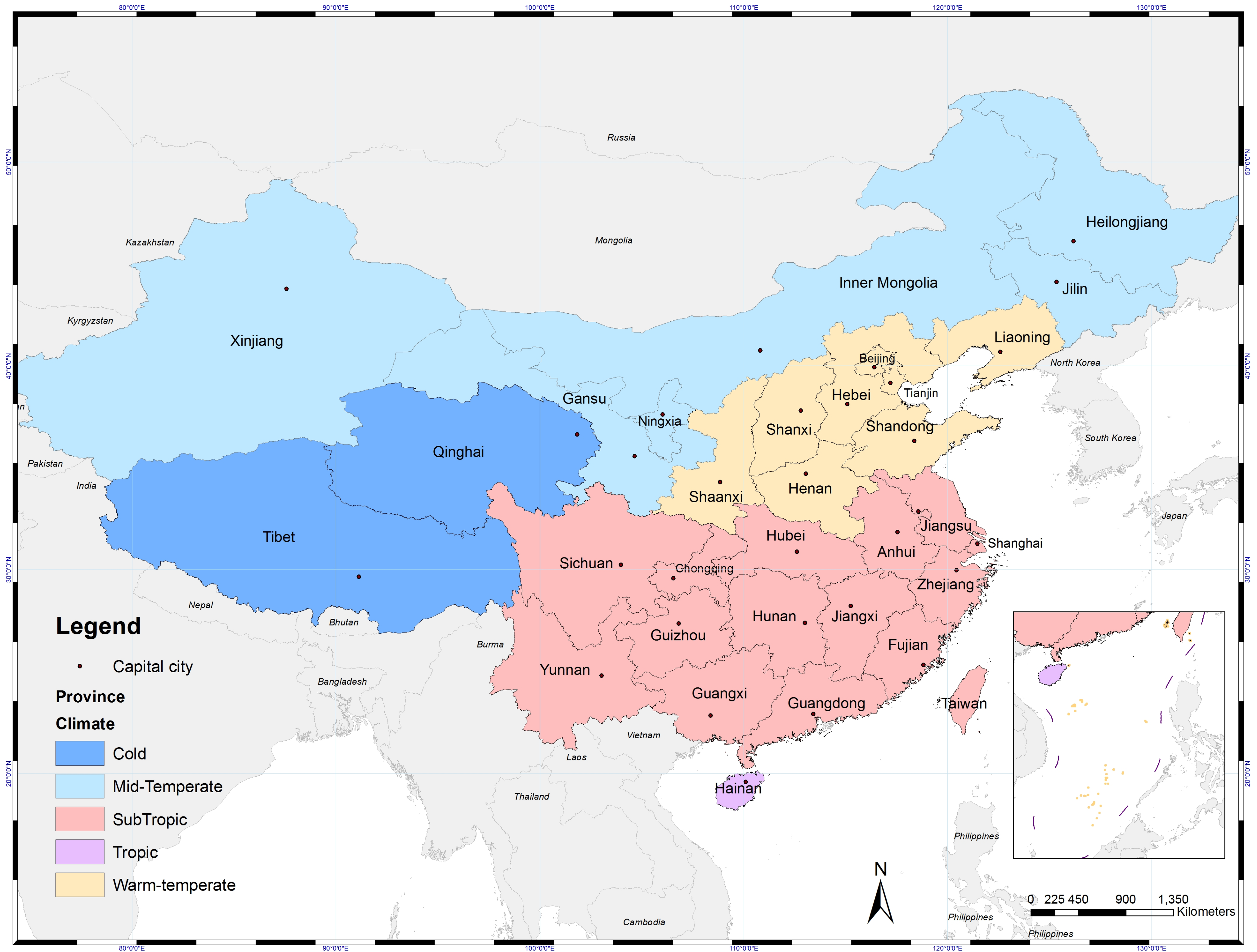

Supplement: Additional file 3: Figure S1. — The general climate of each province in mainland China. The data is from the China Meteorological Administration (http://www.cma.gov.cn/). [file 12916_2015_336_MOESM3_ESM.pdf]

**A Imported case**

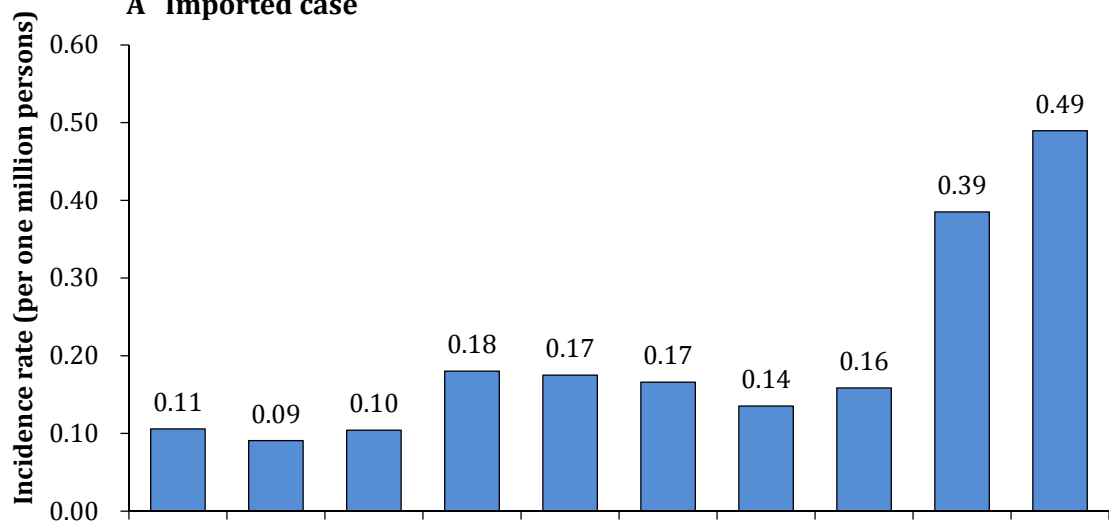

**B Indigenous case**

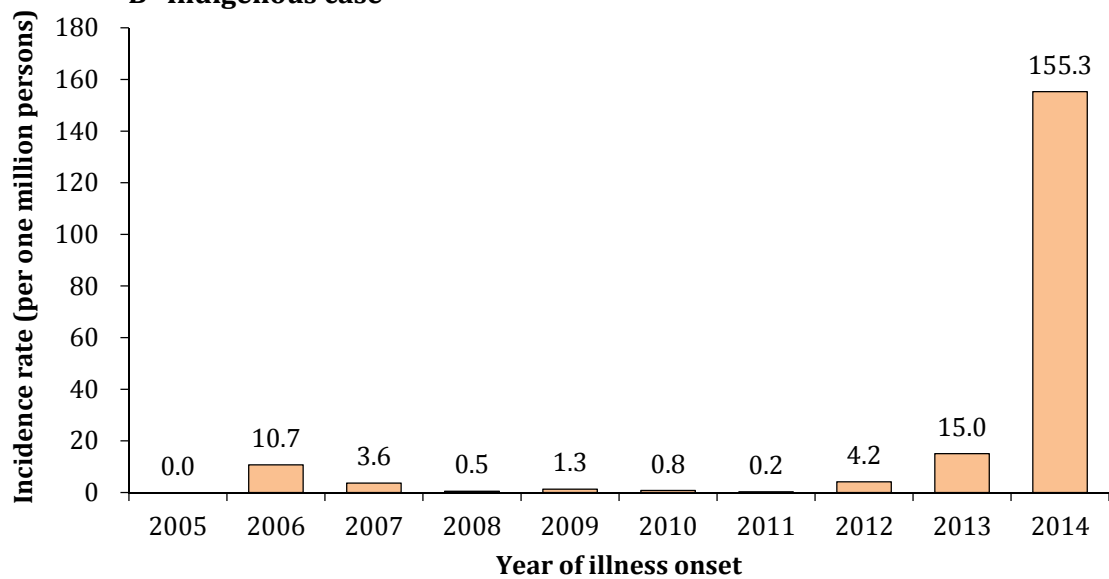

Supplement: Additional file 8: Figure S2. — The morbidity of imported (N = 2,061) and indigenous (N = 53,053) dengue cases in mainland China, 2005-2014. Panel A: The morbidity of imported cases per one million persons of affected provinces at each year-end. Panel B: The morbidity of indigenous cases per one million persons of affected provinces at each year-end. [file 12916_2015_336_MOESM8_ESM.pdf]

**A Imported case**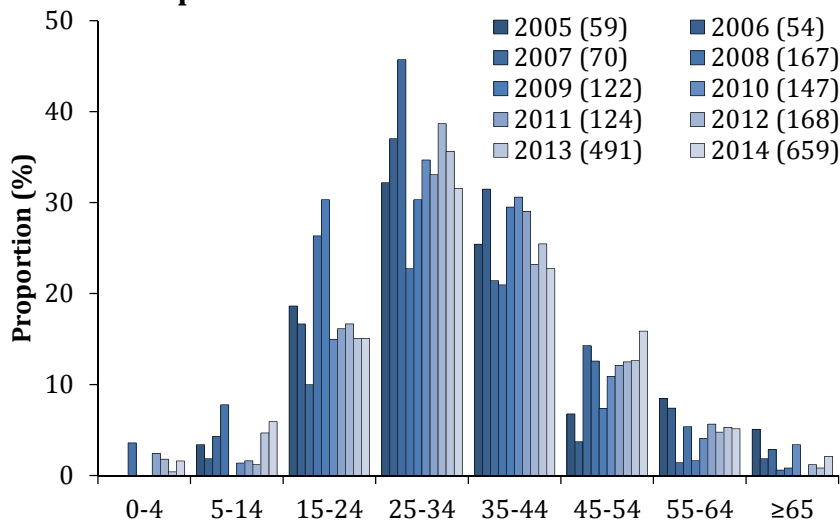**B Indigenous case**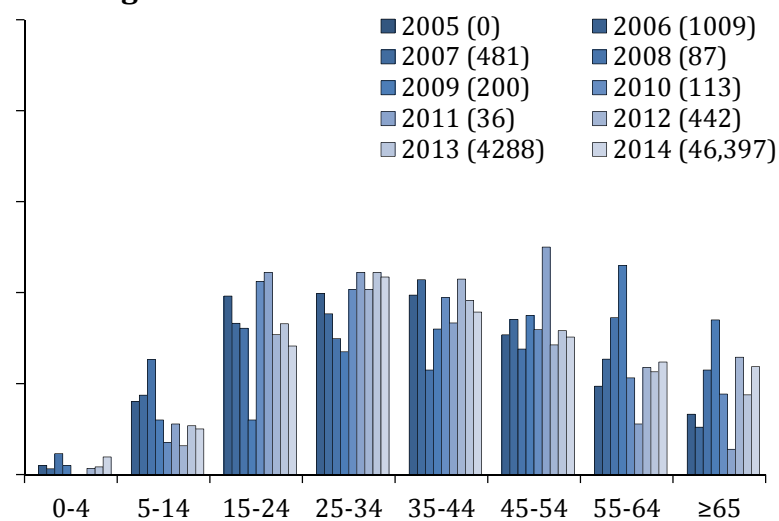**C Imported case**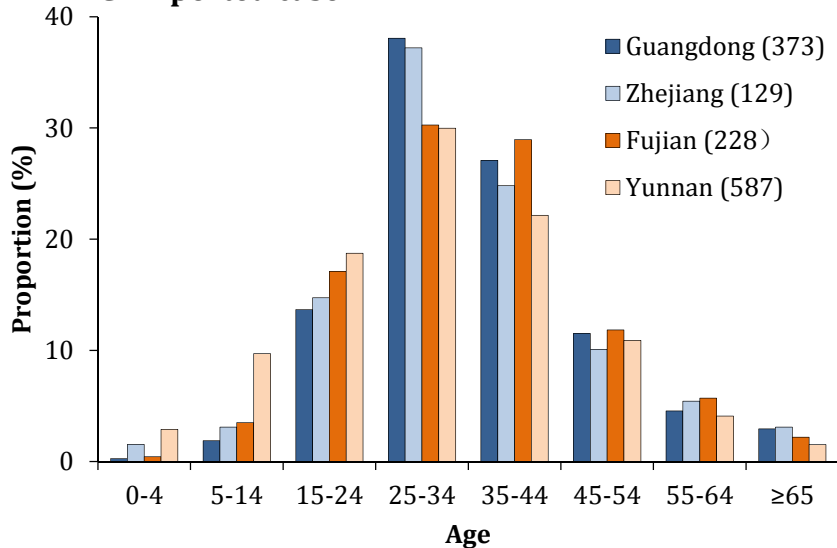**D Indigenous case**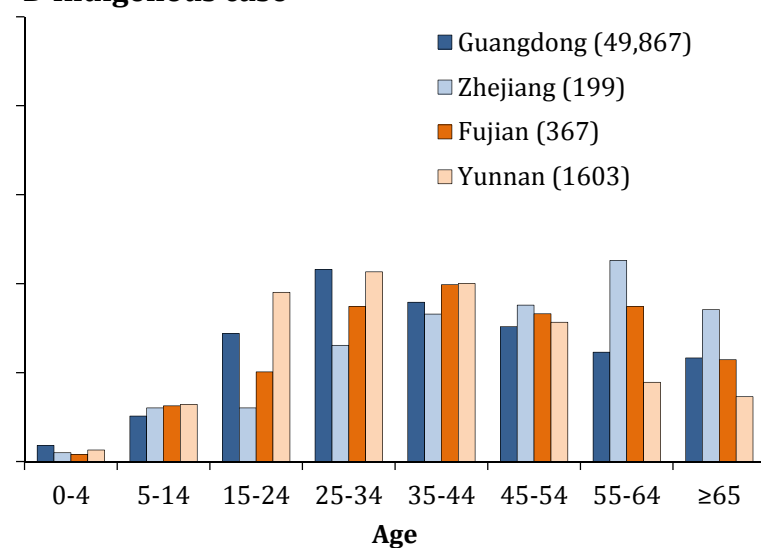

Supplement: Additional file 9: Figure S3. — The age distribution of imported (N = 2,061) and indigenous (N = 53,053) dengue cases by year and province. Panel A: The proportion of imported cases by age and year. Panel B: The proportion of indigenous cases by age and year. Panel C: The proportion of imported cases by age and top four provinces reported cases. Panel D: The proportion of indigenous cases by age and top four provinces reported cases. The number of cases is shown in parentheses in the legend. [file 12916_2015_336_MOESM9_ESM.pdf]
