# Supplementary material for: The changing epidemiology of dengue in China, 1990-2014: a descriptive analysis of 25 years of nationwide surveillance data
Source: BMC Med. 2015 Apr 28;13:100. doi: 10.1186/s12916-015-0336-1 (PMC4431043; doi:10.1186/s12916-015-0336-1)
Supplement: Additional file 4: Table S3. — Summary of the geography and climate of each province in mainland China. [file 12916_2015_336_MOESM4_ESM.pdf]

Table S3. Summary of the geography and climate of each province in mainland China.

No.	Province	Zone code	Climate [a]	Inland or coastal province	Northern or southern	Adjacent country	Capital city	Latitude [b]	Longitude [b]
1	Heilongjiang	230000	Mid-Temperate	Inland	Northern	Russia	Harbin	46.1138	126.185
2	Jilin	220000	Mid-Temperate	Inland	Northern	Russia and North Korea	Changchun	44.1156	125.352
3	Xinjiang	650000	Mid-Temperate	Inland	Northern	Russia, Mongolia, Kazakhstan, Kyrgyzstan, Tajikistan, Afghanistan, Pakistan and India	Urumqi	43.7878	87.574
4	Inner Mongolia	150000	Mid-Temperate	Inland	Northern	Russia and Mongolia	Huhhot	40.7632	110.82
5	Liaoning	210000	Warm-temperate	Coastal	Northern	North Korea	Shenyang	40.6843	122.589
6	Beijing	110000	Warm-temperate	Inland	Northern	None	Beijing	39.94	116.41
7	Tianjin	120000	Warm-temperate	Coastal	Northern	None	Tianjin	39.16	117.2
8	Hebei	130000	Warm-temperate	Coastal	Northern	None	Shijiazhuang	38.1269	115.078
9	Shanxi	140000	Warm-temperate	Inland	Northern	None	Taiyuan	37.8098	112.8
10	Ningxia	640000	Mid-Temperate	Inland	Northern	None	Yinchuan	37.6234	106.026
11	Qinghai	630000	Cold	Inland	Northern	None	Xining	36.6401	101.835
12	Shandong	370000	Warm-temperate	Coastal	Northern	None	Jinan	36.313	118.368
13	Gansu	620000	Mid-Temperate	Inland	Northern	Mongolia	Lanzhou	35.5751	104.657
14	Henan	410000	Warm-temperate	Inland	Northern	None	Zhengzhou	34.707	113.058
15	Shaanxi	610000	Warm-temperate	Inland	Northern	None	Xi'an	34.3038	108.849
16	Jiangsu	320000	SubTropic	Coastal	Southern	None	Nanjing	32.8614	118.575
17	Anhui	340000	SubTropic	Inland	Southern	None	Hefei	31.8527	117.543
18	Shanghai	310000	SubTropic	Coastal	Southern	None	Shanghai	31.28	121.46
19	Hubei	420000	SubTropic	Inland	Southern	None	Wuhan	30.8781	112.606

No.	Province	Zone code	Climate [a]	Inland or coastal province	Northern or southern	Adjacent country	Capital city	Latitude [b]	Longitude [b]
20	Sichuan	510000	SubTropic	Inland	Southern	None	Chengdu	30.2459	103.978
21	Zhejiang	330000	SubTropic	Coastal	Southern	None	Hangzhou	29.9769	120.444
22	Tibet	540000	Cold	Inland	Northern	India, Bhutan, Nepal, Myanmar and Pakistan	Lhasa	29.65	91.13
23	Chongqing	500000	SubTropic	Inland	Southern	None	Chongqing	29.59	106.55
24	Jiangxi	360000	SubTropic	Inland	Southern	None	Nanchang	28.2274	115.261
25	Hunan	430000	SubTropic	Inland	Southern	None	Changsha	27.3878	113.006
26	Guizhou	520000	SubTropic	Inland	Southern	None	Guiyang	27.3627	106.816
27	Fujian	350000	SubTropic	Coastal	Southern	None	Fuzhou	25.337	118.827
28	Yunnan	530000	SubTropic	Inland	Southern	Vietnam, Laos and Myanmar	Kunming	24.8119	103.034
29	Guangdong	440000	SubTropic	Coastal	Southern	None	Guangzhou	22.9286	113.414
30	Guangxi	450000	SubTropic	Coastal	Southern	Vietnam	Nanning	22.85	108.37
31	Hainan	460000	Tropic	Coastal	Southern	None	Haikou	19.5855	110.101

Note: [a] The general climate of each province, which is available on the website of China Meteorological Administration (http://www.cma.gov.cn/). [b] The latitude and longitude of capital city of each province.
