# Supplementary material for: The changing epidemiology of dengue in China, 1990-2014: a descriptive analysis of 25 years of nationwide surveillance data
Source: BMC Med. 2015 Apr 28;13:100. doi: 10.1186/s12916-015-0336-1 (PMC4431043; doi:10.1186/s12916-015-0336-1)
Supplement: Additional file 5: Table S4. — Demographic and epidemiologic characteristics of dengue cases from 2005 to 2014. [file 12916_2015_336_MOESM5_ESM.pdf]

Table S4. Demographic and epidemiologic characteristics of dengue cases from 2005 to 2014.

Characteristics	Total (n=55,114)	Imported cases (n=2,061)	Indigenous cases (n=53,053)
Type of cases			
Lab-confirmed case	41783 (75.8%)	1746 (84.7%)	40037 (75.5%)
Probable case	13331 (24.2%)	315 (15.3%)	13016 (24.5%)
Gender			
Female	27611 (50.1%)	687 (33.3%)	26924 (50.7%)
Male	27503 (49.9%)	1374 (66.7%)	26129 (49.3%)
Age			
Median (yrs, range)	39 (0.01, 107)	32 (0.5, 80)	39 (0.01, 107)
Age group			
0-4	988 (1.8%)	24 (1.2%)	964 (1.8%)
5-14	2814 (5.1%)	86 (4.2%)	2728 (5.1%)
15-24	8012 (14.5%)	347 (16.8%)	7665 (14.4%)
25-34	12156 (22.1%)	687 (33.3%)	11469 (21.6%)
35-44	10089 (18.3%)	517 (25.1%)	9572 (18%)
45-54	8323 (15.1%)	264 (12.8%)	8059 (15.2%)
55-64	6614 (12%)	103 (5%)	6511 (12.3%)
65 and above	6118 (11.1%)	33 (1.6%)	6085 (11.5%)
Nationality			
Chinese	54608 (99.1%)	1571 (76.2%)	53037 (100%)
Foreigner	506 (0.9%)	490 (23.8%)	16 (0.03%)
Hospitalization			
Yes	6408 (11.6%)	172 (8.3%)	6236 (11.8%)
No	10611 (19.3%)	54 (2.6%)	10557 (19.9%)
Unknown	38095 (69.1%)	1835 (89%)	36260 (68.3%)
Year of onset			
2005	59 (0.1%)	59 (2.9%)	0 (0)
2006	1063 (1.9%)	54 (2.6%)	1009 (1.9%)
2007	551 (1%)	70 (3.4%)	481 (0.9%)
2008	254 (0.5%)	167 (8.1%)	87 (0.2%)
2009	322 (0.6%)	122 (5.9%)	200 (0.4%)
2010	260 (0.5%)	147 (7.1%)	113 (0.2%)
2011	160 (0.3%)	124 (6%)	36 (0.1%)
2012	610 (1.1%)	168 (8.2%)	442 (0.8%)
2013	4779 (8.7%)	491 (23.8%)	4288 (8.1%)
2014	47056 (85.4%)	659 (32%)	46397 (87.5%)
Month of onset			
January	44 (0.1%)	44 (2.1%)	0 (0)
February	50 (0.1%)	50 (2.4%)	0 (0)

March	61 (0.1%)	61 (3%)	0 (0)
April	73 (0.1%)	72 (3.5%)	1 (0.002%)
May	110 (0.2%)	110 (5.3%)	0 (0)
June	135 (0.2%)	113 (5.5%)	22 (0%)
July	584 (1.1%)	158 (7.7%)	426 (0.8%)
August	3363 (6.1%)	290 (14.1%)	3073 (5.8%)
September	21824 (39.6%)	399 (19.4%)	21425 (40.4%)
October	26278 (47.7%)	507 (24.6%)	25771 (48.6%)
November	2434 (4.4%)	182 (8.8%)	2252 (4.2%)
December	158 (0.3%)	75 (3.6%)	83 (0.2%)
Median of time delay (days, range)			
From illness onset to diagnosis	5 (0, 196)	6 (0, 196)	5 (0, 141)
From diagnosis to report [a]	0.3 (-196, 31)	0.2 (-196, 31)	0.3 (-122, 15)
From illness onset to report	6 (0.3, 82)	6 (0.3, 82)	6 (0.3, 64)
Serotype of Dengue virus			
I	373 (0.7%)	11 (0.5%)	362 (0.7%)
II	42 (0.1%)	2 (0.1%)	40 (0.1%)
III	16 (0.03%)	3 (0.1%)	13 (0.02%)
IV	2 (0.004%)	2 (0.1%)	0 (0)
Unknown	54681 (99.2%)	2043 (99.1%)	52638 (99.2%)
Case imported from other province in China			
Yes	235 (0.4%)	235 (11.4%)	0 (0)
No	54879 (99.6%)	1826 (88.6%)	53053 (100%)

Note: Data are presented as no. (%) of patients unless otherwise indicated. [a] The negative number of the median from diagnosis to report means that case was reported by physician as a suspected dengue patient to the surveillance system before diagnosed as a probable or laboratory confirmed dengue cases.
