# Supplementary material for: The changing epidemiology of dengue in China, 1990-2014: a descriptive analysis of 25 years of nationwide surveillance data
Source: BMC Med. 2015 Apr 28;13:100. doi: 10.1186/s12916-015-0336-1 (PMC4431043; doi:10.1186/s12916-015-0336-1)
Supplement: Additional file 6: Table S5. — Demographic and epidemiologic characteristics of imported dengue cases by year from 2005 to 2014. [file 12916_2015_336_MOESM6_ESM.pdf]

Table S5. Demographic and epidemiologic characteristics of imported dengue cases by year from 2005 to 2014.

Characteristics	Total (n=2061)	2005-2013 (n=1402)	2014 (n=659)
Type of cases			
Lab-confirmed case	1746 (84.7%)	1177 (84%)	569 (86.3%)
Probable case	315 (15.3%)	225 (16%)	90 (13.7%)
Gender			
Female	687 (33.3%)	452 (32.2%)	235 (35.7%)
Male	1374 (66.7%)	950 (67.8%)	424 (64.3%)
Age			
Median (yrs, range)	32 (0.5, 80)	32 (0.6, 80)	33 (0.5, 76)
Age group			
0-4	24 (1.2%)	14 (1%)	10 (1.5%)
5-14	86 (4.2%)	48 (3.4%)	38 (5.8%)
15-24	347 (16.8%)	252 (18%)	95 (14.4%)
25-34	687 (33.3%)	478 (34.1%)	209 (31.7%)
35-44	517 (25.1%)	363 (25.9%)	154 (23.4%)
45-54	264 (12.8%)	160 (11.4%)	104 (15.8%)
55-64	103 (5%)	68 (4.9%)	35 (5.3%)
65 and above	33 (1.6%)	19 (1.4%)	14 (2.1%)
Nationality			
Chinese	1571 (76.2%)	1066 (76%)	505 (76.6%)
Foreigner	490 (23.8%)	336 (24%)	154 (23.4%)
Hospitalization			
Yes	172 (8.3%)	116 (8.3%)	56 (8.5%)
No	54 (2.6%)	19 (1.4%)	35 (5.3%)
Unknown	1835 (89%)	1267 (90.4%)	568 (86.2%)
Month of onset			
January	44 (2.1%)	28 (2%)	16 (2.4%)
February	50 (2.4%)	40 (2.9%)	10 (1.5%)
March	61 (3%)	44 (3.1%)	17 (2.6%)
April	72 (3.5%)	63 (4.5%)	9 (1.4%)
May	110 (5.3%)	82 (5.8%)	28 (4.2%)
June	113 (5.5%)	82 (5.8%)	31 (4.7%)
July	158 (7.7%)	131 (9.3%)	27 (4.1%)
August	290 (14.1%)	243 (17.3%)	47 (7.1%)
September	399 (19.4%)	259 (18.5%)	140 (21.2%)
October	507 (24.6%)	262 (18.7%)	245 (37.2%)
November	182 (8.8%)	117 (8.3%)	65 (9.9%)
December	75 (3.6%)	51 (3.6%)	24 (3.6%)
Median of time delay (days, range)			

From illness onset to diagnosis	6 (0, 196)	6 (0, 196)	5 (0.3, 140)
From diagnosis to report [a]	0.2 (-196, 31)	0.3 (-196, 31)	0.1 (-137, 1)
From illness onset to report	6 (0.3, 82)	6 (0.4, 82)	5 (0.3, 48)
Serotype of Dengue virus			
I	11 (0.5%)	9 (0.6%)	2 (0.3%)
II	2 (0.1%)	1 (0.1%)	1 (0.2%)
III	3 (0.1%)	3 (0.2%)	0 (0)
IV	2 (0.1%)	2 (0.1%)	0 (0)
Unknown	2008 (99.1%)	1387 (98.9%)	621 (99.5%)
Case imported from other province in China			
Yes	235 (11.4%)	8 (0.6%)	227 (34.4%)
No	1826 (88.6%)	1394 (99.4%)	432 (65.6%)

Note: Data are presented as no. (%) of patients unless otherwise indicated. [a] The negative number of the median from diagnosis to report means that case was reported by physician as a suspected dengue patient to the surveillance system before diagnosed as a probable or laboratory confirmed dengue cases.
