# Supplementary material for: The changing epidemiology of dengue in China, 1990-2014: a descriptive analysis of 25 years of nationwide surveillance data
Source: BMC Med. 2015 Apr 28;13:100. doi: 10.1186/s12916-015-0336-1 (PMC4431043; doi:10.1186/s12916-015-0336-1)
Supplement: Additional file 7: Table S6. — Demographic and epidemiologic characteristics of indigenous dengue cases by year from 2005 to 2014. [file 12916_2015_336_MOESM7_ESM.pdf]

Table S6. Demographic and epidemiologic characteristics of indigenous dengue cases by year from 2005 to 2014.

Characteristics	Total (n=53053)	2005-2013 (n=6656)	2014 (n=46,397)
Type of cases			
Lab-confirmed case	40037 (75.4%)	5466 (82.1%)	34571 (74.5%)
Probable case	13016 (24.6%)	1190 (17.9%)	11826 (25.5%)
Gender			
Female	26924 (50.7%)	3505 (52.7%)	23419 (50.5%)
Male	26129 (49.3%)	3151 (47.3%)	22978 (49.5%)
Age			
Median (yrs, range)	39 (0.01, 107)	38 (0.2, 96)	39 (0.01, 107)
Age group			
0-4	964 (1.8%)	56 (0.8%)	908 (2%)
5-14	2728 (5.1%)	396 (5.9%)	2332 (5%)
15-24	7665 (14.4%)	1115 (16.8%)	6550 (14.1%)
25-34	11469 (21.6%)	1399 (21%)	10070 (21.7%)
35-44	9572 (18%)	1288 (19.4%)	8284 (17.9%)
45-54	8059 (15.2%)	1052 (15.8%)	7007 (15.1%)
55-64	6511 (12.3%)	770 (11.6%)	5741 (12.4%)
65 and above	6085 (11.5%)	580 (8.7%)	5505 (11.9%)
Nationality			
Chinese	53037 (100%)	6656 (100%)	46381 (100%)
Foreigner	16 (0%)	0 (0)	16 (0%)
Hospitalization			
Yes	6202 (11.7%)	749 (11.3%)	5453 (11.8%)
No	10537 (19.9%)	155 (2.3%)	10382 (22.4%)
Unknown	36164 (68.4%)	5752 (86.4%)	30412 (65.8%)
Month of onset			
January	0 (0)	0 (0)	0 (0)
February	0 (0)	0 (0)	0 (0)
March	0 (0)	0 (0)	0 (0)
April	1 (0.002%)	1 (0.02%)	0 (0)
May	0 (0)	0 (0)	0 (0)
June	22 (0.04%)	4 (0.1%)	18 (0.04%)
July	426 (0.8%)	170 (2.6%)	256 (0.6%)
August	3073 (5.8%)	1161 (17.4%)	1912 (4.1%)
September	21425 (40.4%)	2333 (35.1%)	19092 (41.1%)
October	25771 (48.6%)	2431 (36.5%)	23340 (50.3%)
November	2252 (4.2%)	552 (8.3%)	1700 (3.7%)
December	83 (0.2%)	4 (0.1%)	79 (0.2%)
Median of time delay (days, range)			

From illness onset to diagnosis	5 (0, 141)	6 (0, 141)	5 (0, 95)
From diagnosis to report [a]	0.3 (-122, 15)	0.1 (-122, 15)	0.3 (-84, 8)
From illness onset to report	6 (0.3, 64)	6 (0.4, 62)	5 (0.3, 64)
Serotype of Dengue virus			
I	362 (0.7%)	61 (0.9%)	301 (0.6%)
II	40 (0.1%)	1 (0%)	39 (0.1%)
III	13 (0.02%)	13 (0.2%)	0 (0)
Unknown	52638 (99.2%)	6581 (98.9%)	46057 (99.3%)

Note: Data are presented as no. (%) of patients unless otherwise indicated. [a] The negative number of the median from diagnosis to report means that case was reported by physician as a suspected dengue patient to the surveillance system before diagnosed as a probable or laboratory confirmed dengue cases.
