# Supplementary material for: The changing epidemiology of dengue in China, 1990-2014: a descriptive analysis of 25 years of nationwide surveillance data
Source: BMC Med. 2015 Apr 28;13:100. doi: 10.1186/s12916-015-0336-1 (PMC4431043; doi:10.1186/s12916-015-0336-1)
Supplement: Additional file 10: Figure S4. — The serotype distribution of imported (N = 18) and indigenous (N = 415) dengue cases by province, 2009-2014. Panel A: Serotype distribution of imported cases. Among 18 imported cases with serotype data, all four serotypes were reported: DENV-1 (11 cases), DENV-2 (2), DENV-3 (3), and DENV-4 (2) during 2009-2014. Panel B: Serotype distribution of indigenous cases. Data on serotypes were only available for 415 indigenous cases during 2005-2014: 362 (87.2%) cases with DENV-1 in Guangdong during 2011-2014, 40 (9.6%) DENV-2 in Guangdong during 2013-2014, and 13 (3.1%) DENV-3 in Zhejiang in 2009 and Guangdong during 2012-2013. [file 12916_2015_336_MOESM10_ESM.pdf]

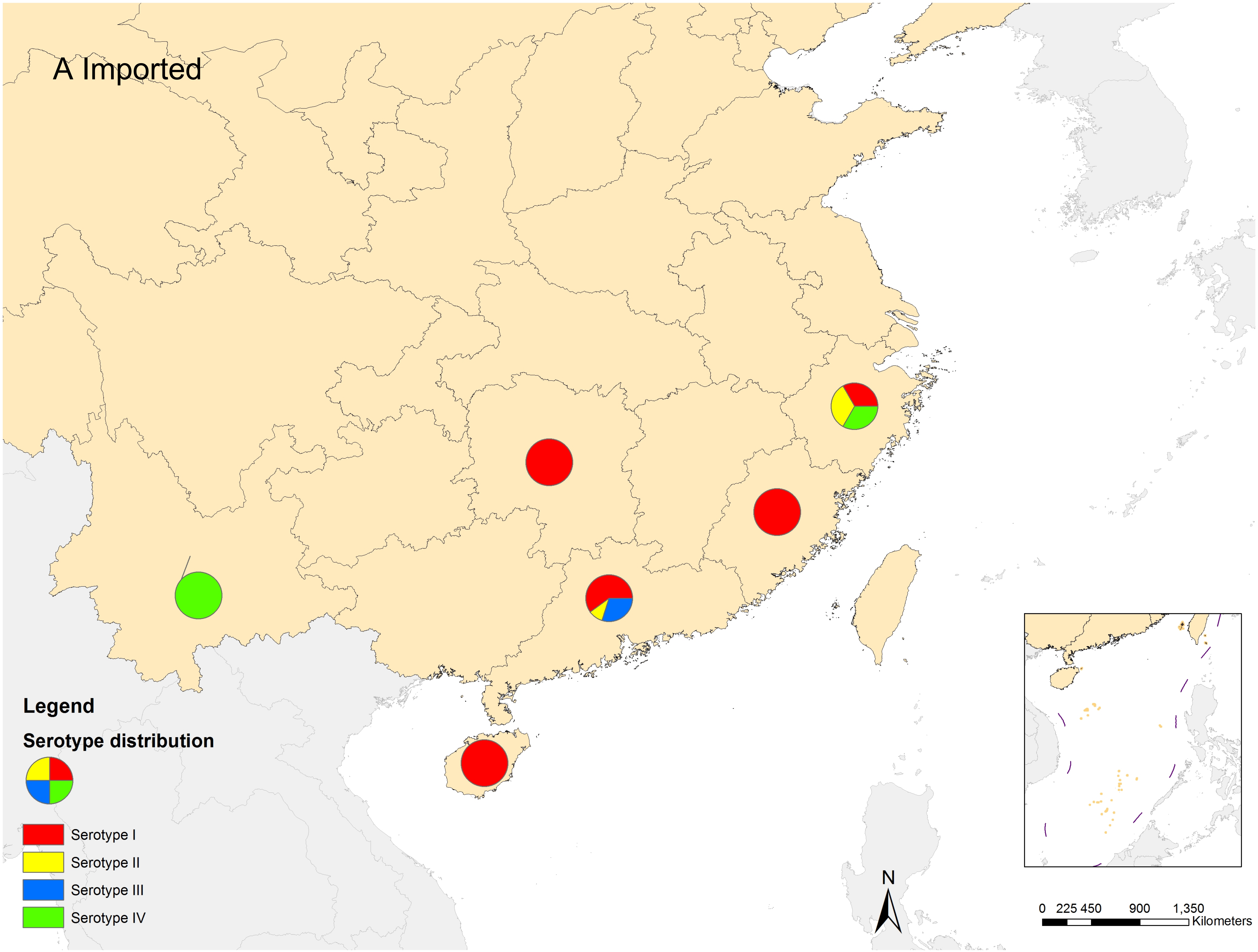

A Imported
Legend
Serotype distribution
Serotype I
Serotype II
Serotype III
Serotype IV
N
0 225 450 900 1,350
Kilometers

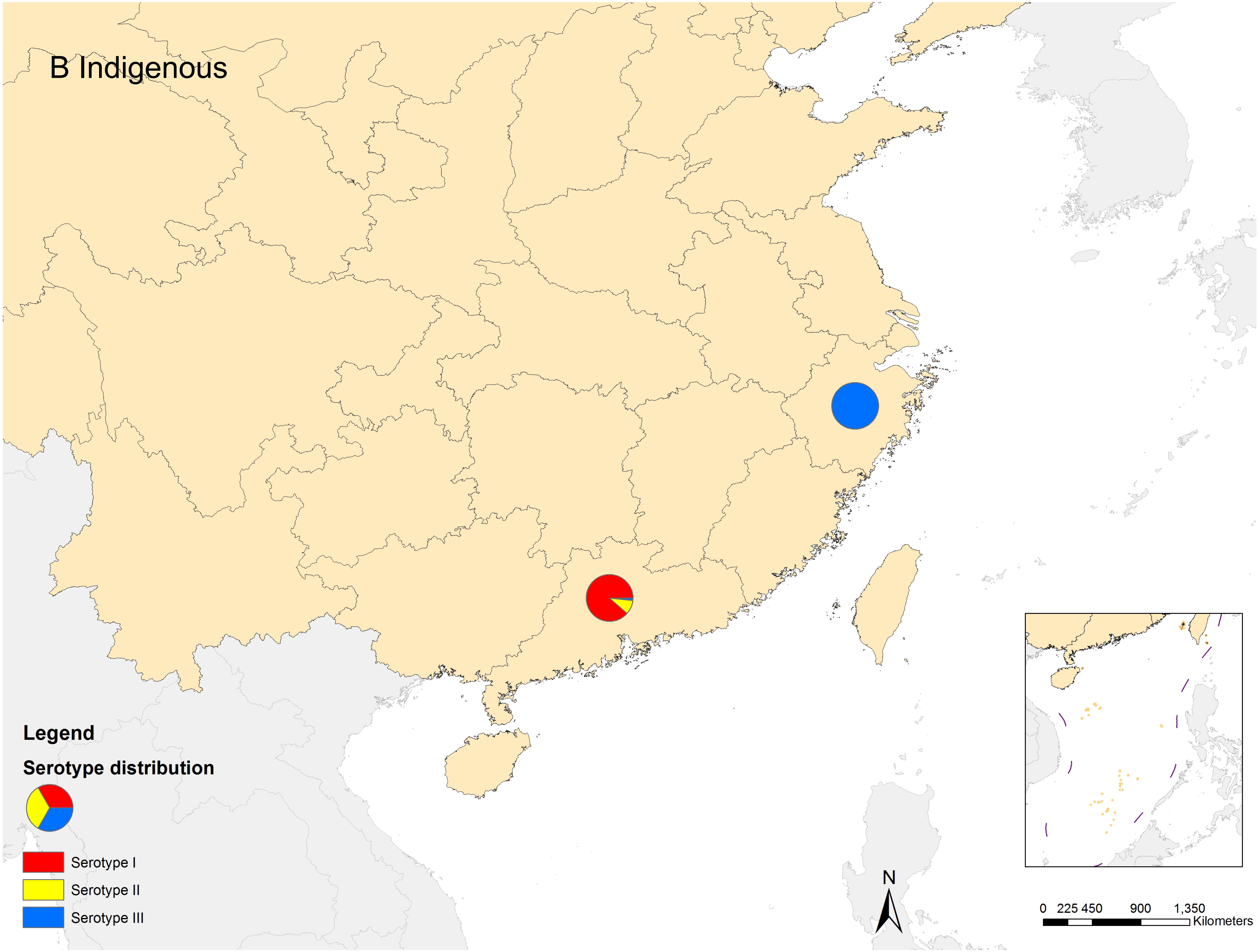
B Indigenous
Legend
Serotype distribution
Serotype I
Serotype II
Serotype III
N
0 225 450 900 1,350
Kilometers
