# Supplementary material for: The changing epidemiology of dengue in China, 1990-2014: a descriptive analysis of 25 years of nationwide surveillance data
Source: BMC Med. 2015 Apr 28;13:100. doi: 10.1186/s12916-015-0336-1 (PMC4431043; doi:10.1186/s12916-015-0336-1)
Supplement: Additional file 11: Figure S5. — The geographic distribution of dengue cases by year in mainland China, 2005-2012. [file 12916_2015_336_MOESM11_ESM.pdf]

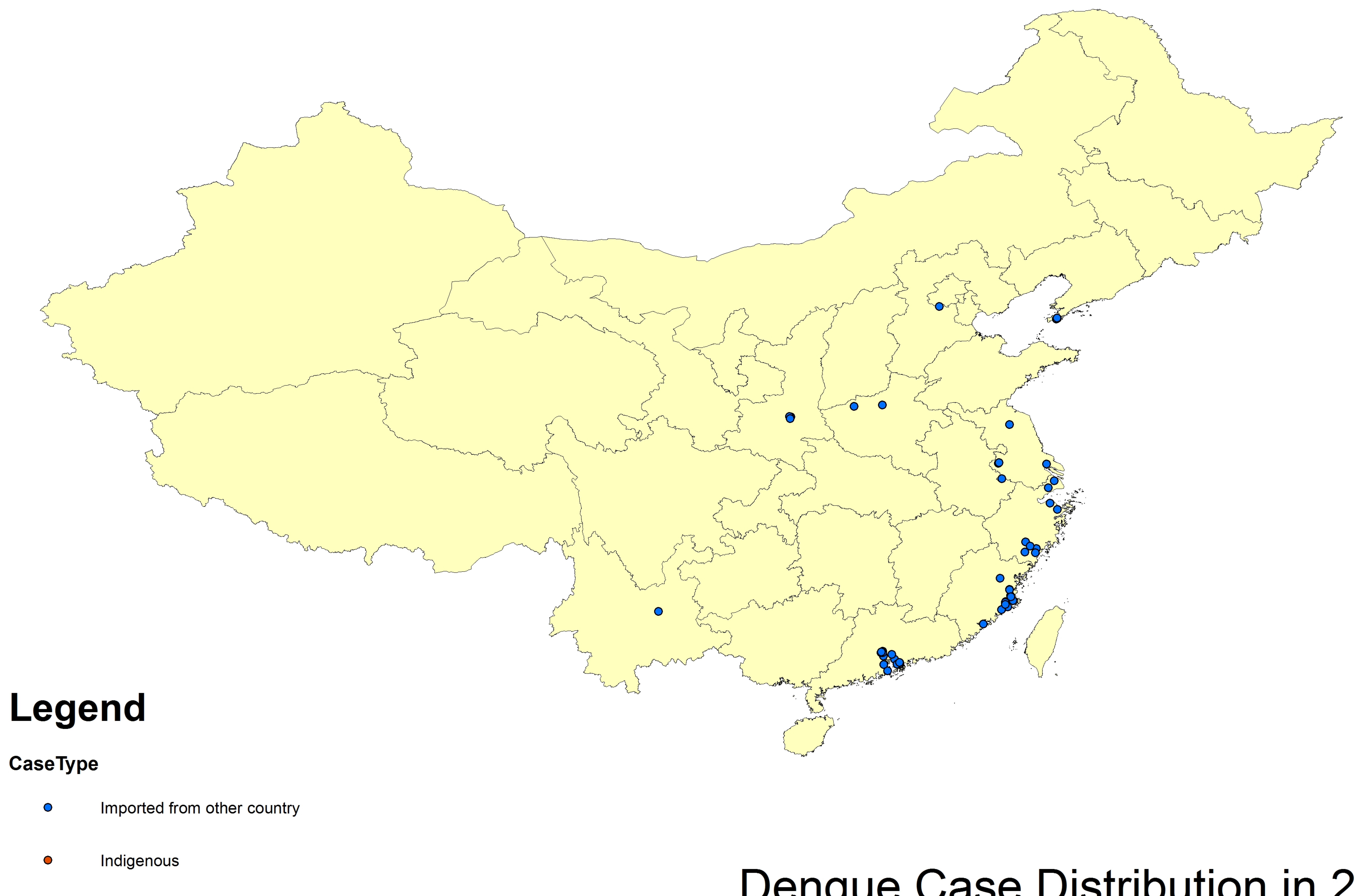

Dengue Case Distribution in 2005

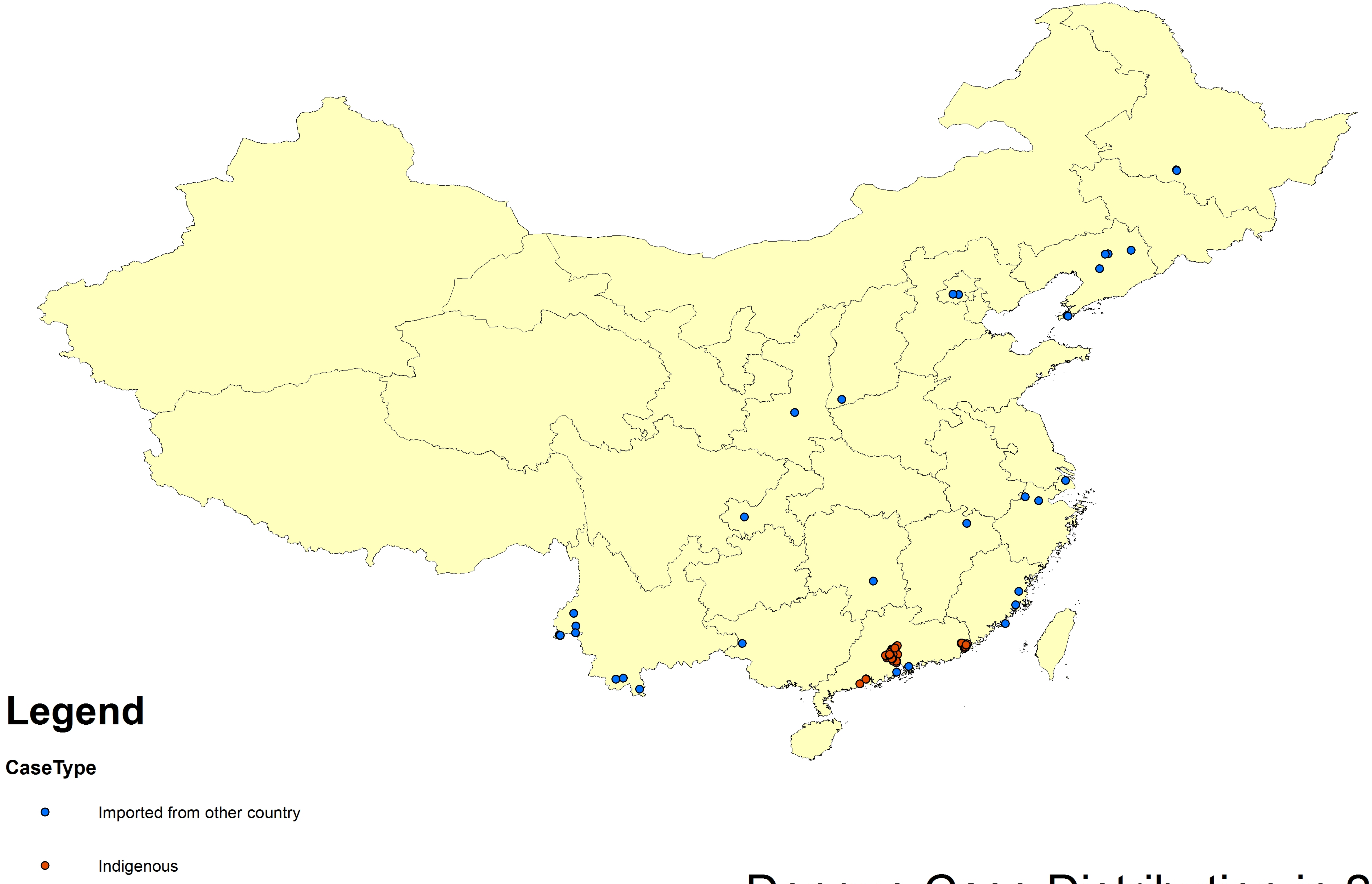

Dengue Case Distribution in 2006

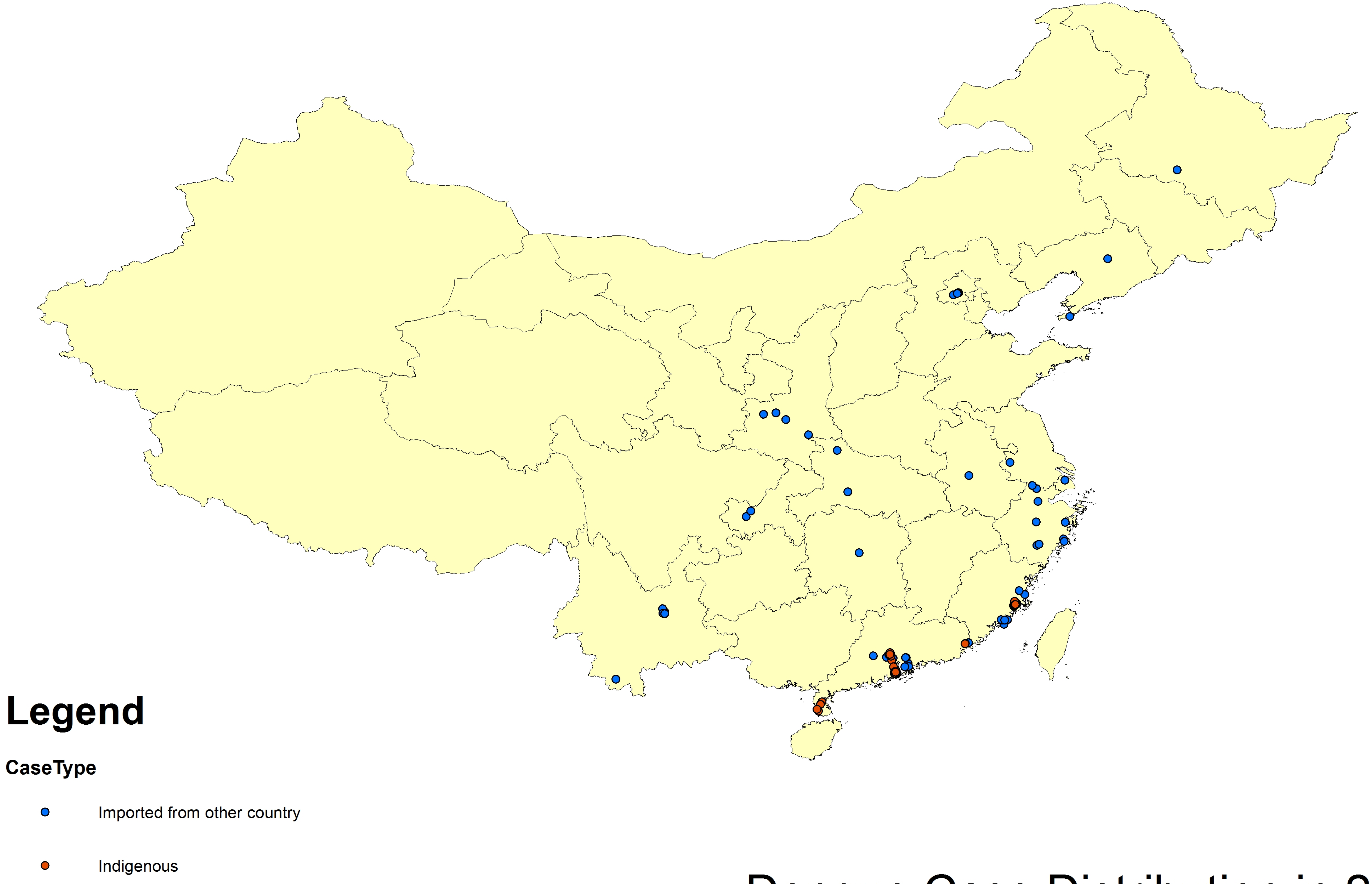

Dengue Case Distribution in 2007

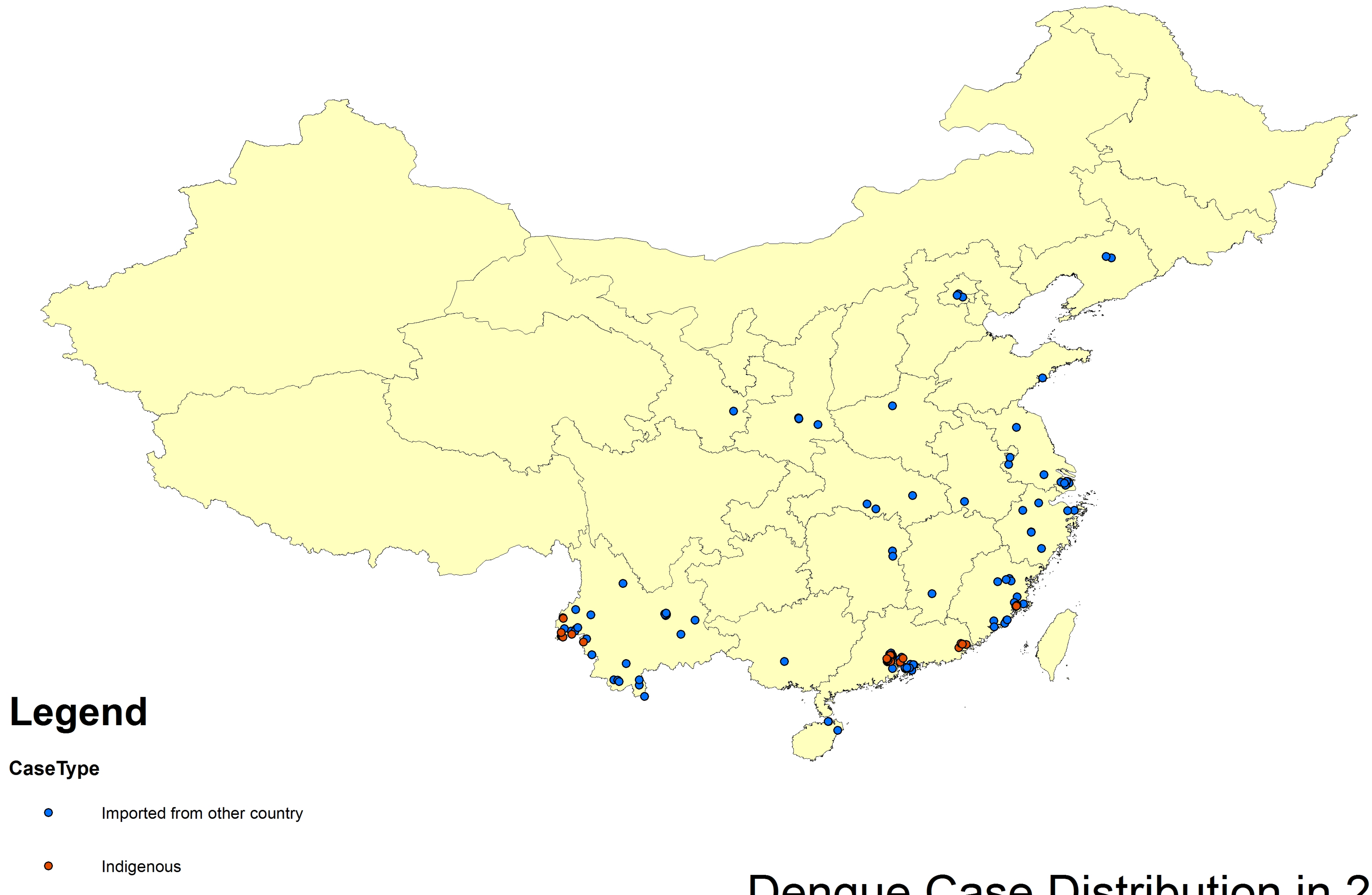

Dengue Case Distribution in 2008

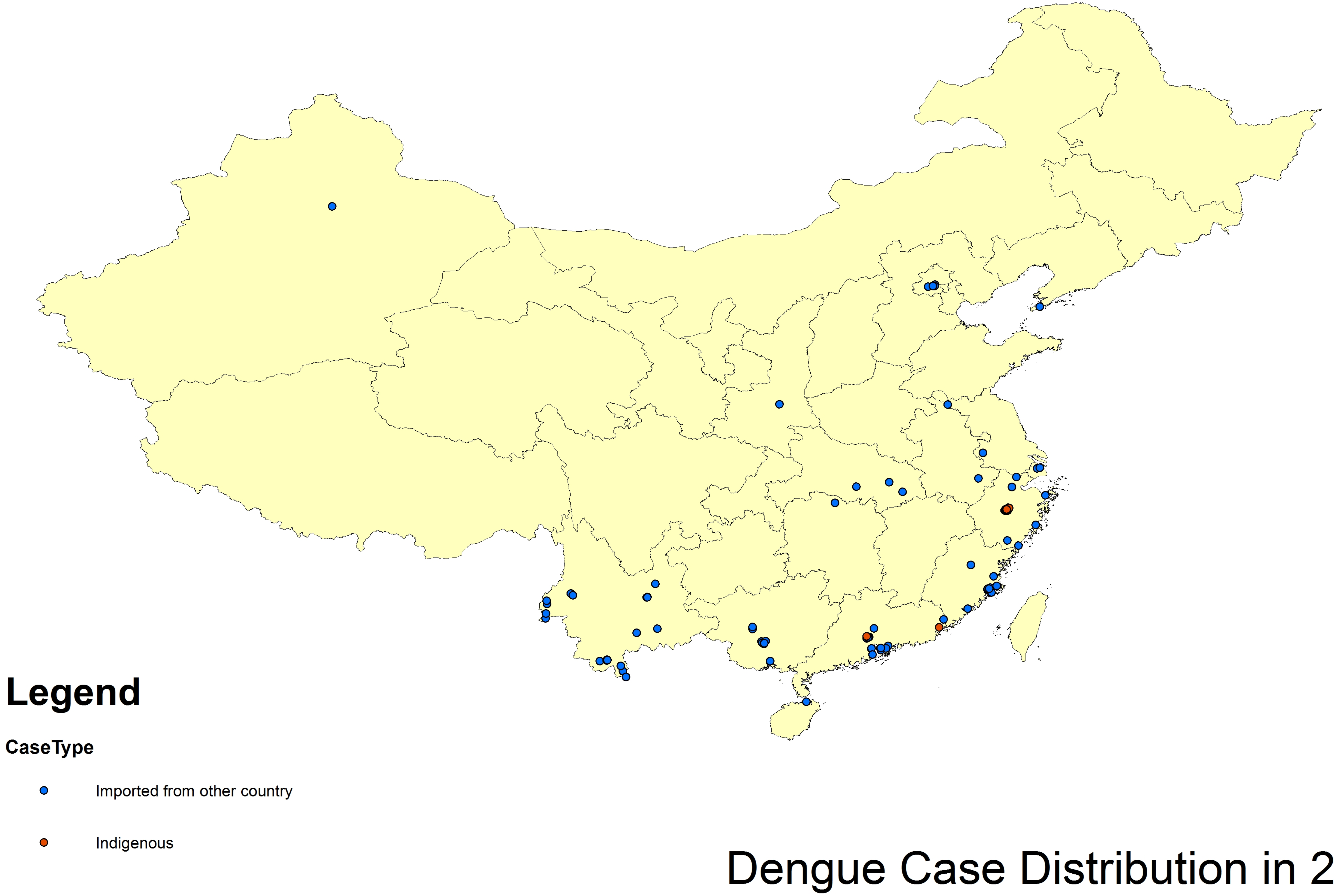

Dengue Case Distribution in 2009

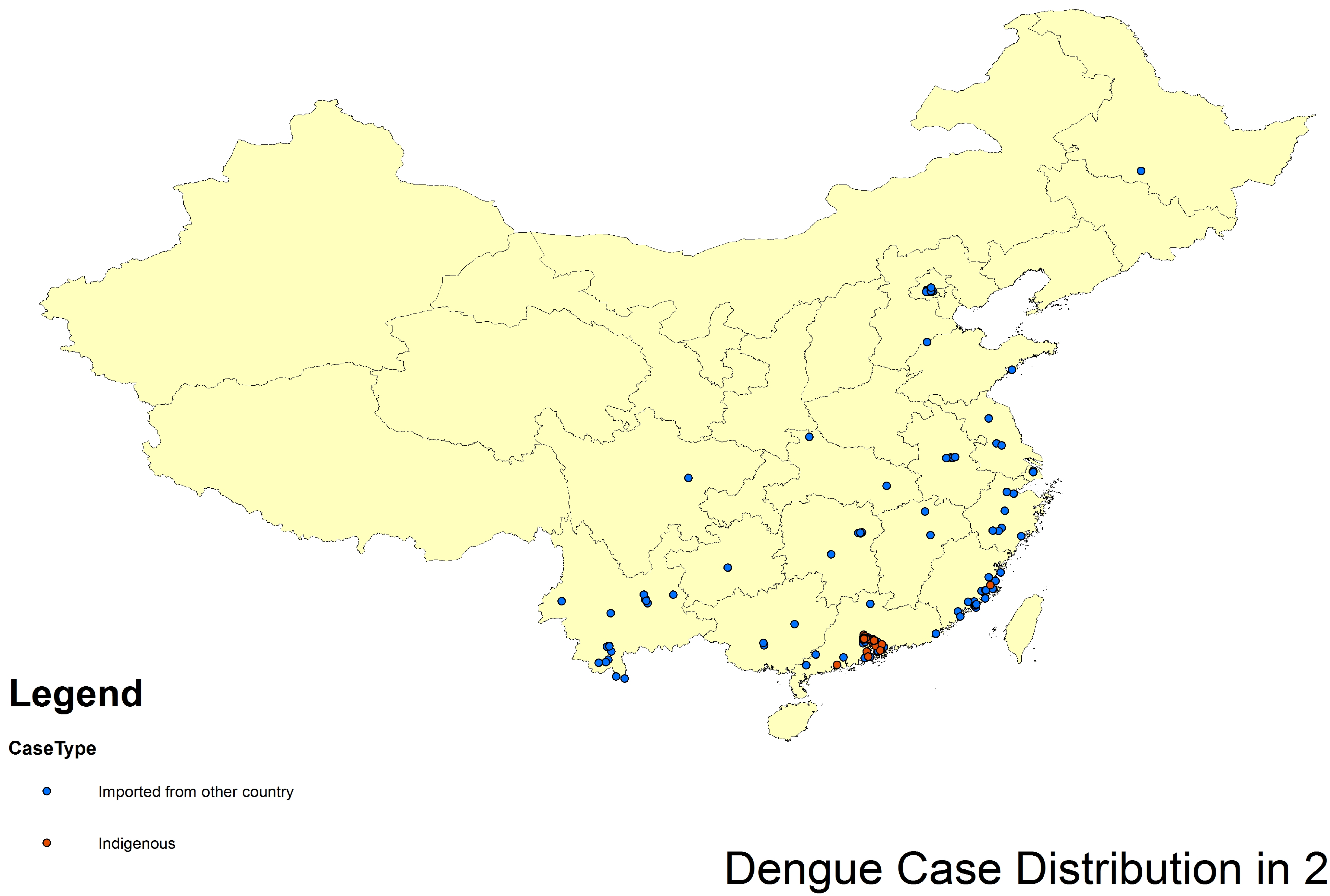

Dengue Case Distribution in 2010

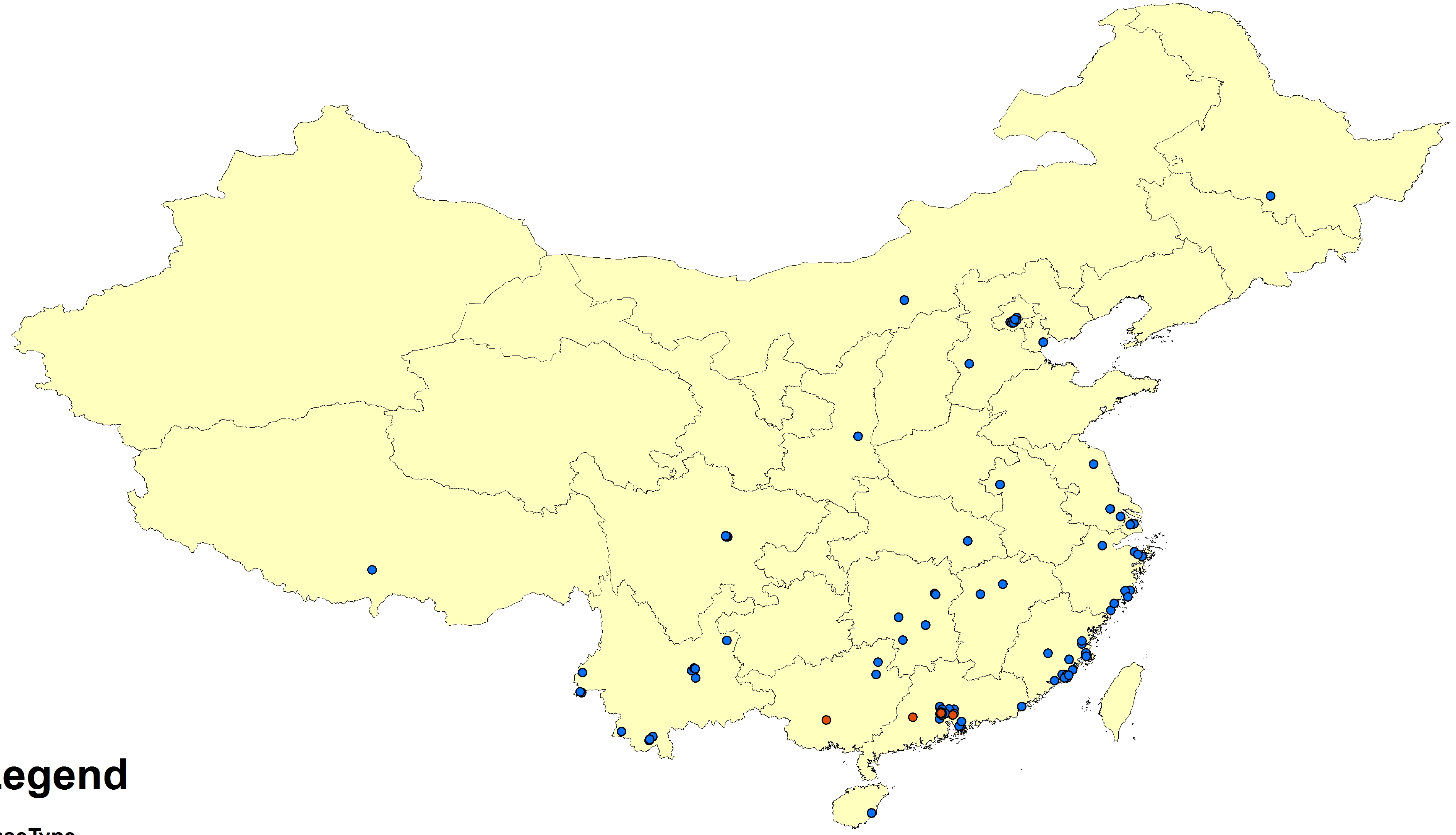

Legend

CaseType

- Imported from other country
- Indigenous

Dengue Case Distribution in 2011

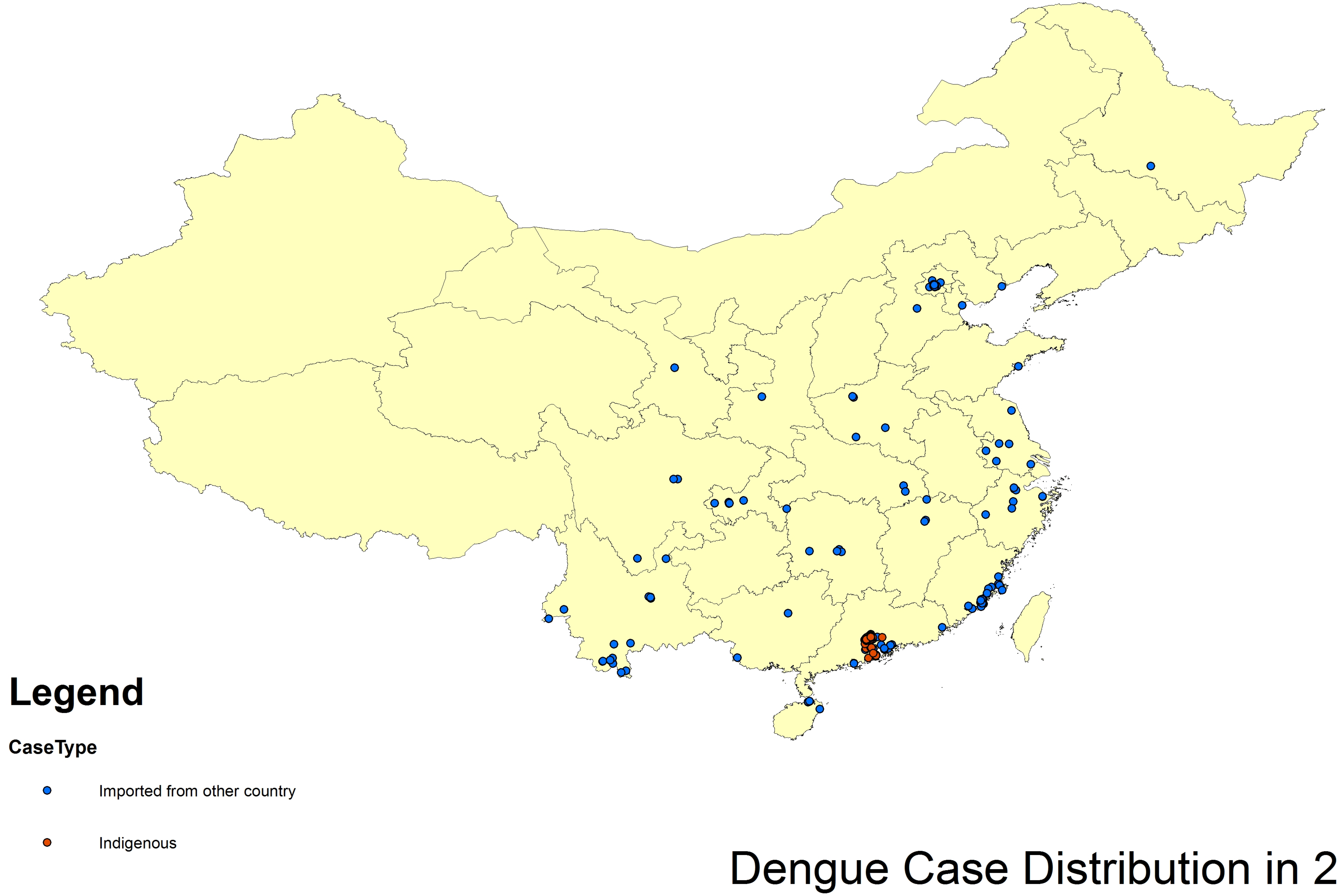

Dengue Case Distribution in 2012
